# Cuproptosis-mediated stemness inhibition by a self-assembled herbal nanoplatform overcomes chemoresistance in ovarian cancer

**DOI:** 10.1016/j.mtbio.2026.103440

**Published:** 2026-07-09

**Authors:** Shanshan Liu, Yichun Huang, Fanchen Yan, Hailong Tian, Bowen Li, Yaying Zhang, Huili Zhu, Weihua Tong, Canhua Huang

**Affiliations:** a School of Health and Rehabilitation, Chengdu University of Traditional Chinese Medicine, Chengdu, 611137, China; b Department of Surgical Oncology, The First Affiliated Hospital of Kunming Medical University, Kunming, 650032, China; c School of Basic Medical Sciences, Chengdu University of Traditional Chinese Medicine, Chengdu, 611137, China; d Department of Biotherapy, Oxidative Stress Research Center, Cancer Center and State Key Laboratory of Biotherapy, West China Hospital, Sichuan University, Chengdu, Sichuan, 610041, China; e Department of Reproductive Medicine, Key Laboratory of Birth Defects and Related Diseases of Women and Children of Ministry of Education, West China Second University Hospital of Sichuan University, Chengdu, Sichuan, 610041, China; f Obstetrics and Gynecology Center, The First Hospital of Jilin University, Changchun, Jilin, 130012, China; g Frontiers Medical Center, Tianfu Jincheng Laboratory, Chengdu, 610041, China

**Keywords:** Ovarian cancer, Cuproptosis, Cancer stem cells, Chemoresistance, Shikonin

## Abstract

Ovarian cancer remains difficult to treat due to the persistence of chemoresistant cancer stem-like cells (CSCs), which are major drivers of tumor relapse. Herein, we engineered a hyaluronic acid-functionalized nanoplatform (HS-Cu@DOX) via the assembly of a shikonin–copper (SKN-Cu) coordination complex with hyaluronic acid, followed by electrostatic loading of doxorubicin (DOX), enabling tumor-targeted co-delivery of both therapeutic agents. This nanoplatform disrupts intracellular redox homeostasis to induce cuproptosis while concurrently suppressing CSC stemness through downregulation of key pluripotency-associated transcription factors (e.g., SOX2, OCT4, and NANOG). By depleting intracellular glutathione (GSH) and impairing antioxidant defenses, HS-Cu@DOX disrupts the oxidative stress resilience of CSCs, thereby restoring their sensitivity to DOX-induced apoptosis. Consequently, this strategy enhances DOX-mediated cytotoxicity and reduces systemic toxicity while maintaining potent antitumor efficacy. Collectively, our findings demonstrate that combining cuproptosis induction with CSC stemness suppression represents a promising strategy for overcoming chemoresistance, highlighting HS-Cu@DOX as a potential therapeutic candidate for recurrent ovarian cancer.

## Highlights

• A hyaluronic acid-coated nanoparticle (HS-Cu@DOX) co-delivers a shikonin-copper complex and doxorubicin. This design uniquely integrates active CD44-targeting with controlled release to achieve synergistic chemosensitization.
• Copper-induced cuproptosis and GSH depletion foster the suppression of cancer stem cells.
• Shikonin-mediated stemness inhibition and cuproptosis combined foster the reversal of chemoresistance and delay of tumor recurrence.

## Graphical abstract

**Figure:**
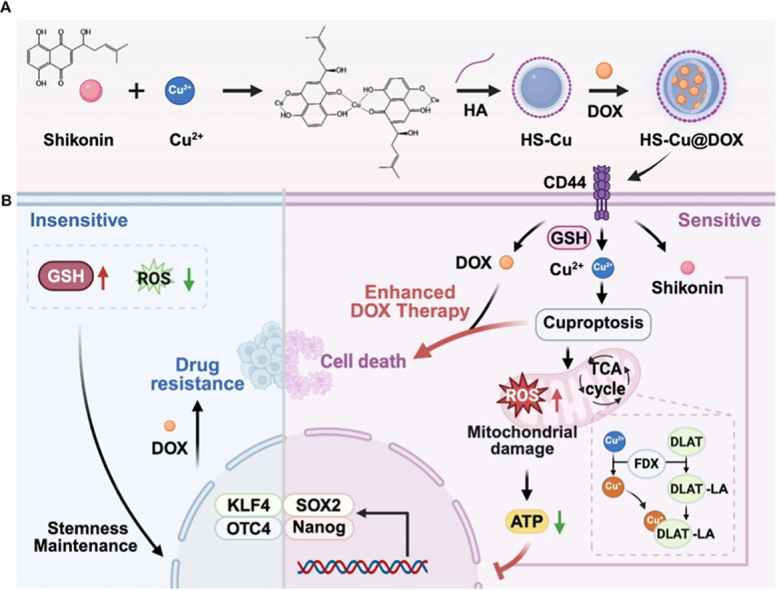

## 1 Introduction

Ovarian cancer is one of the most aggressive gynecologic malignancies, characterized by dismal long-term survival rates [1]. The unfavorable prognosis is primarily attributable to the frequent development of chemoresistance and subsequent disease recurrence [2]. Mounting evidence suggests that cancer stem-like cells (CSCs) play a pivotal role in this process. CSCs constitute a highly tumorigenic subpopulation characterized by enhanced self-renewal capacity, efficient DNA damage repair, and robust antioxidant defense mechanisms [3,4]. These CSCs exhibit elevated intracellular glutathione (GSH) levels, which efficiently neutralize reactive oxygen species (ROS) generated by conventional chemotherapeutic agents, such as doxorubicin (DOX) [5,6]. This enhanced redox buffering capacity protects CSCs from oxidative damage, promotes their survival under therapeutic stress, and ultimately contributes to treatment resistance and disease relapse [7,8]. Therefore, targeting these adaptive mechanisms represents a promising strategy for overcoming chemoresistance and improving therapeutic outcomes in ovarian cancer.

Transition metal ions, particularly copper, have recently considerable attention owing to their unique redox properties and emerging potential in cancer therapy [9]. Copper can participate in Fenton-like reactions to enhance oxidative stress and, importantly, directly deplete intracellular glutathione (GSH) through the formation of stable coordination complexes [10]. Beyond redox regulation, copper ions can induce cuproptosis, a recently identified form of regulated cell death characterized by mitochondrial proteotoxic stress [11,12]. This process is initiated by the binding of copper to lipoylated proteins within the tricarboxylic acid (TCA) cycle, resulting in aberrant protein aggregation, destabilization of iron–sulfur cluster proteins, and subsequent mitochondrial dysfunction [13]. Importantly, this metabolic disruption not only induces tumor cell death but also impairs the stem-like properties of CSCs, which rely heavily on mitochondrial metabolism to maintain self-renewal and cellular plasticity [14,15]. By disrupting key metabolic pathways and inducing proteotoxic stress, copper-based therapeutic strategies have the potential to target both bulk tumor cells and treatment-resistant CSCs.

Shikonin, a natural naphthoquinone compound derived from traditional medicinal plants, has attracted considerable interest for its broad anticancer properties [16,17]. It has been reported to suppress the expression of key stemness-associated factors, including Nanog, OCT4, and SOX2, thereby impairing the self-renewal and tumor-initiating capacities of CSCs [18,19]. However, the clinical translation of shikonin remains limited by several challenges. Its hydrophobic naphthoquinone structure results in poor aqueous solubility and low bioavailability. In addition, its small-molecule nature leads to nonspecific biodistribution, limited tumor accumulation, and undesirable uptake by normal tissues. Furthermore, shikonin monotherapy exhibits limited efficacy against drug-resistant CSCs, whereas dose escalation is associated with increased hepatotoxicity [20]. To address these limitations, shikonin was coordinated with copper ions through its intrinsic metal-chelating capability, forming a stable shikonin–copper (SKN-Cu) complex. This complex not only preserves the stemness-suppressive activity of shikonin but also enhances its antitumor efficacy through GSH depletion and cuproptosis induction, thereby enabling a multifaceted therapeutic strategy against chemoresistant ovarian cancer cells.

**Scheme 1:**
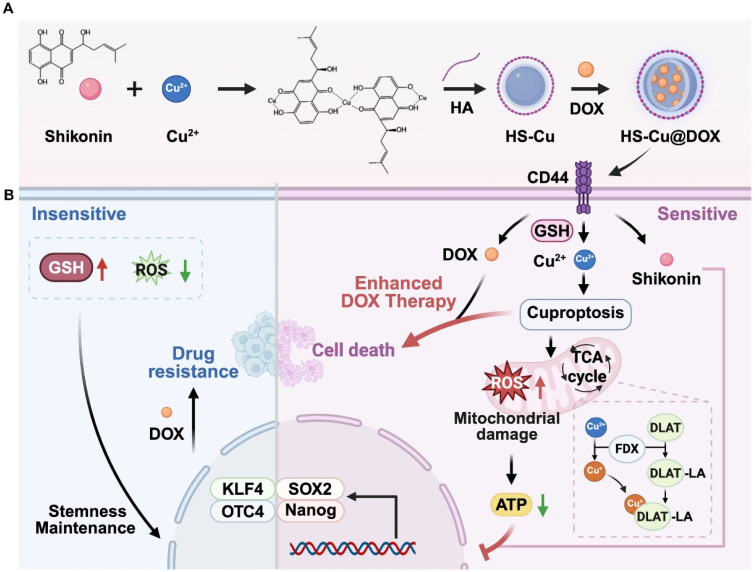
A) The preparation of HS-Cu@DOX. B) The underlying mechanism of HS-Cu@DOX for therapeutic resistance. Created with BioRender.com.

In this study, we developed a self-assembled nanoparticle system, termed HS-Cu@DOX (Scheme 1), by first coordinating shikonin with copper ions to form a stable SKN-Cu complex, subsequently assembling the complex with hyaluronic acid (HA), and finally loading doxorubicin (DOX) through electrostatic interactions. The HA-modified nanoparticles were designed to actively target CD44-overexpressing ovarian cancer cells. We hypothesized that HS-Cu@DOX would synergistically integrate the stemness-suppressive activity of shikonin, the GSH-depleting and cuproptosis-inducing effects of copper ions, and the chemotherapeutic efficacy of DOX, thereby overcoming CSC-associated chemoresistance in ovarian cancer. Both *in vitro* and *in vivo* studies demonstrated that HS-Cu@DOX effectively ameliorated DOX resistance and significantly delayed tumor recurrence. This nanoparticle-based approach provides an innovative strategy for sensitizing ovarian tumors to chemotherapy through the simultaneous induction of cuproptosis and suppression of CSC defense mechanisms, offering a promising therapeutic avenue for recurrent disease.

## 2 Results and discussion

### 2.1 Synthesis strategy and characterization of HS-Cu@DOX

**Fig. 1:**
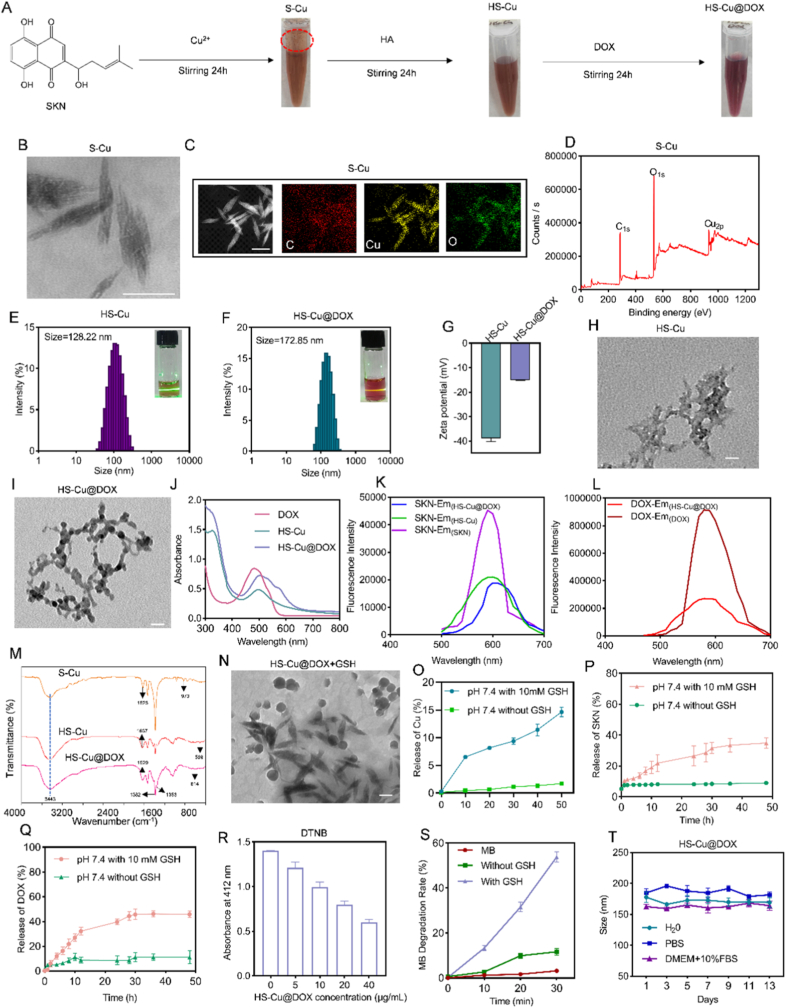
Preparation and characterization of HS-Cu@DOX. A) Preparation of HS-Cu@DOX. B) TEM images of S-Cu. Scale: 100 nm. C) Elemental mapping of S-Cu, including bright field, C, Cu, and O. Scale: 100 nm. D) XPS of S-Cu. E, F) Tyndall effect and size distribution of (E) HS-Cu, and (F) HS-Cu@DOX. G) The zeta potential of HS-Cu and HS-Cu@DOX (n = 3). H, I) TEM images of (H) HS-Cu, and (I) HS-Cu@DOX. Scale: 100 nm. J) UV−vis spectra of DOX, HS-Cu, and HS-Cu@DOX in water. K) Fluorescence emission of SKN in ethanol: free state, with HS-Cu, and with HS-Cu@DOX. L) Fluorescence emission of DOX in ddH_2_O: free state and with HS-Cu@DOX. M) FTIR spectra of S-Cu, HS-Cu and HS-Cu@DOX. N) The TEM image of HS-Cu@DOX after GSH treatment. O) Time-dependent cumulative Cu^2+^ release from HS-Cu@DOX nanoparticles, quantified by ICP-OES (n = 3). P, Q) The release behavior of (P) SKN and (Q) DOX (n = 3). R) DTNB assay measurement of HS-Cu@DOX-induced glutathione depletion *in vitro* (n = 3). S) OH-dependent MB degradation induced by HS-Cu@DOX with/without GSH. Data shown as Mean ± SD (n = 3). T) Size change of HS-Cu@DOX in ddH_2_O, DMEM and PBS solution over 13 days (n = 3).

HS-Cu@DOX was synthesized via a stepwise assembly process (Fig. 1A). Initially, a hydrophobic shikonin-copper complex (S-Cu) was formed through coordination between SKN and Cu^2+^ at pH 8 to 9. Transmission electron microscopy (TEM) revealed that the S-Cu complex exhibited an aggregated needle-like clustered structure (Fig. 1B). Elemental mapping analysis via energy-dispersive X-ray spectroscopy (EDS) further confirmed the presence and distribution of carbon (C), copper (Cu), and oxygen (O) throughout the S-Cu complex (Fig. 1C and Fig. S1A–B, Supporting Information). Furthermore, X-ray photoelectron spectroscopy (XPS) analysis showed a characteristic Cu 2p peak at 934.26 eV, confirming the successful incorporation of copper into the S-Cu complex (Fig. 1D).

To improve the aqueous dispersibility of S-Cu, hyaluronic acid (HA) was assembled onto the complex to form water-dispersible HS-Cu nanoparticles. Optimization of the HA/SKN mass ratio revealed that a ratio of 1:1 yielded nanoparticles with an optimal hydrodynamic diameter of 128.22 nm while minimizing SKN usage. Deviations from this ratio (e.g., 2:1) resulted in significant aggregation (Fig. S1C–F, Supporting Information). This optimized formulation exhibited a pronounced Tyndall effect, indicating good colloidal stability, while nanoparticle tracking analysis (NTA) further confirmed the size distribution of HS-Cu nanoparticles (Fig. 1E). Next, DOX was loaded onto HS-Cu via electrostatic interactions to yield HS-Cu@DOX. Both the Tyndall effect and NTA confirmed that HS-Cu@DOX possessed a mean particle size of 172.85 nm (Fig. 1F), which is within the optimal range for the EPR effect–mediated passive tumor targeting. Zeta potential measurements revealed that HS-Cu nanoparticles exhibited a negative surface charge (−38.9 mV), which shifted to −14.9 mV upon DOX loading in HS-Cu@DOX (Fig. 1G), indicating successful electrostatic loading of DOX. TEM analysis further revealed a distinct morphological transition: the S-Cu complex exhibited needle-like clustered structures, whereas the HA-coated HS-Cu nanoparticles formed a uniformly dispersed cross-linked network (Fig. 1H). The enhanced aqueous stability conferred by HA coating was visually corroborated by comparative digital images of S-Cu and HS-Cu aqueous solutions shown in Fig. 1A. While HS-Cu@DOX largely retained this network architecture, the appearance of spherical particulates on its surface further suggested successful DOX loading (Fig. 1I).

Spectroscopic analyses, including UV-vis, fluorescence, and FTIR spectroscopy, were used to investigate molecular interactions and confirm successful drug loading. UV-vis spectra showed characteristic peaks of free SKN at 515/560 nm in ethanol (Fig. S1G, Supporting Information). The HS-Cu complex exhibited a ligand-metal charge transfer (LMCT) band at 500 nm, whereas HS-Cu@DOX displayed a modified LMCT band at 500 nm and a shifted SKN absorption peak at 580 nm (Fig. 1J). Importantly, the disappearance of the 480 nm absorption band of free DOX confirmed its incorporation into the nanocomposite. Fluorescence spectroscopy revealed significant quenching and shifting of SKN emission peak in HS-Cu compared with free SKN, providing direct evidence of Cu^2+^-SKN coordination. This quenching effect was also retained in HS–Cu@DOX. Additionally, a substantial decrease in DOX fluorescence intensity in HS-Cu@DOX confirmed successful drug loading and interaction with the HS-Cu carrier (Fig. 1K–L and Figure S1H-I, Supporting Information). FTIR spectroscopy spectra further confirmed the successful assembly of S-Cu, HS-Cu, and HS-Cu@DOX (Fig. 1M).

The disassembly of HS-Cu@DOX can be induced by GSH-dependent reduction of Cu^2+^, facilitating therapeutic release for enhanced chemotherapy [21]. As shown in the TEM images, HS-Cu@DOX underwent obvious structural disruption after GSH treatment, indicating the dissociation of the copper-coordinated assembly (Fig. 1N). Consistent with the TEM observations, HS-Cu@DOX exhibited a pronounced change in appearance after GSH treatment, accompanied by a decrease in color intensity and the formation of precipitates, indicating disruption of the nanoparticle structure (Fig. S1J, Supporting Information). Consistently, the appearance of HS-Cu@DOX dispersions changed over time in PBS containing 10 mM GSH, whereas no noticeable changes were observed in PBS without GSH (Fig. S1M–N, Supporting Information), confirming the redox-responsive nature of the nanoplatform. The release profile of Cu^2+^ was quantified using inductively coupled plasma optical emission spectroscopy (ICP-OES). As shown in Fig. 1O, HS-Cu@DOX exhibited sustained Cu^2+^ release in the presence of GSH, indicating gradual dissociation of the copper-coordinated framework. We further evaluated the release profiles of SKN and DOX using standard curves shown in Fig. S1K–L (Supporting Information). Significant release of both drugs was observed in PBS (pH 7.4) containing 10 mM GSH, whereas only minimal leakage was observed in the absence of GSH (Fig. 1P–Q). These results demonstrate the GSH-triggered release behavior of HS-Cu@DOX.

The peroxidase-like activity of copper was exploited to induce GSH- mediated Fenton-like reactions, generating hydroxyl radicals (·OH), and triggering oxidative stress [22,23]. To assess the GSH responsiveness of HS-Cu@DOX, we conducted the 5,5′-dithiobis-(2-nitrobenzoic acid) (DTNB) assay. As depicted in Fig. 1R, increasing concentrations of HS-Cu@DOX induced progressive GSH depletion. The Fenton-like activity of HS-Cu@DOX was further validated using methylene blue (MB) as a probe. MB degradation, indicated by a decrease in absorbance at 664 nm, was most pronounced in the HS-Cu@DOX + GSH group [24], suggesting that HS-Cu@DOX induces oxidative stress and mitochondrial oxidative damage (Fig. 1S).

Finally, the stability of HS-Cu@DOX was evaluated under various physiological conditions, including ddH_2_O, DMEM (10% FBS), and PBS. The hydrodynamic diameter remained consistent for 13 days (Fig. 1T), indicating excellent stability critical for *in vivo* applications. DOX and SKN payloads were quantified using high-performance liquid chromatography (HPLC) and UV-vis spectrophotometry, respectively (Fig. S1O–P, Supporting Information). The loading efficiencies were 26.16% for SKN and 23.00% for DOX in HS-Cu@DOX (Fig. S1Q, Supporting Information). In addition, ICP-OES analysis confirmed efficient copper incorporation into the nanoplatform, with a Cu^2+^ loading efficiency of 13.79% (Fig. S1R, Supporting Information). The high loading efficiencies of SKN, DOX, and Cu^2+^ can be attributed to the unique coordination-driven assembly strategy of the nanoplatform. Specifically, SKN functions not only as a therapeutic agent but also as a structural component of the nanoparticle core through coordination with Cu^2+^, thereby facilitating its incorporation into the nanostructure. The resulting compact SKN-Cu coordination framework enables efficient retention of both SKN and Cu^2+^ within the nanoparticles. Furthermore, electrostatic interactions between DOX and the HS-Cu matrix promote stable DOX incorporation. In addition, HA primarily serves as a surface-stabilizing component rather than a bulky carrier matrix, minimizing the proportion of inactive materials and thereby contributing to the high overall loading capacity of the system.

### 2.2 Cellular internalization and cytotoxic effects of HS-Cu@DOX *in vitro*

**Fig. 2:**
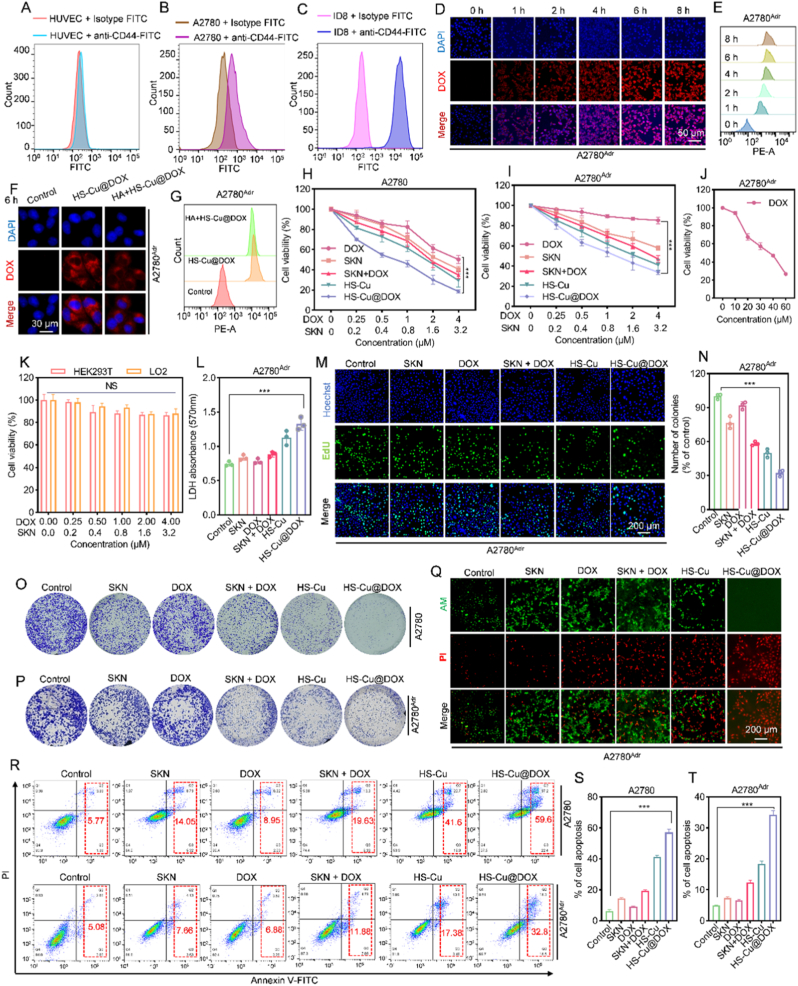
The cellular internalization and cytotoxic effects of HS-Cu@DOX *in vitro*. SKN and DOX concentrations were set as follows: A2780 (0.57 μM SKN, 0.70 μM DOX), ID8 (0.43 μM SKN, 0.52 μM DOX), and A2780^Adr^ (1.13 μM SKN, 1.39 μM DOX), with HS-Cu and HS-Cu@DOX administered at equivalent drug doses. A–C) Representative flow cytometry histograms of CD44 expression on HUVEC, A2780, and ID8 cells, respectively. D, E) Cellular uptake of HS-Cu@DOX in A2780^Adr^ cells was assessed via (D) fluorescence microscopy and (E) flow cytometry. C_DOX_ = 2 μM; Scale bar: 50 μm. F, G) Cellular uptake of Control, HS-Cu@DOX and HA + HS-Cu@DOX in A2780^Adr^ cells after 6 h incubation, as examined by fluorescence microscopy and flow cytometry. Cells were pretreated with HA for 30 min. C_DOX_ = 2 μM; Scale bar: 30 μm. H, I) Cell viability of (H) A2780 and (I) A2780^Adr^ cells following treatment with Control, DOX, SKN, DOX + SKN, HS-Cu and HS-Cu@DOX (n = 3). J) Viability of A2780^Adr^ cells treated with gradient DOX concentrations. K) The viability of HEK293T and LO2 cells following treatment with HS-Cu@DOX at different concentrations (n = 3). L) LDH assay of A2780^Adr^ cells co-cultured with Control, DOX, SKN, DOX + SKN, HS-Cu and HS-Cu@DOX. M) Fluorescent images of EdU labeling test in A2780^Adr^ cells subjected to various treatments (n = 3). Scale bar: 200 μm. N) The statistical analysis for colony formation of A2780^Adr^ cells following treatment with Control, DOX, SKN, DOX + SKN, HS-Cu and HS-Cu@DOX. O) The images for colony formation of A2780 cells following treatment with Control, DOX, SKN, DOX + SKN, HS-Cu and HS-Cu@DOX. P) Representative images of A2780^Adr^ cells after various treatments for colony formation. Q) Fluorescence imaging of AM/PI staining assay in A2780^Adr^ cells with different treatments; scale bar:200 μm. R-T) Flow cytometric analysis and quantification of apoptosis in A2780 and A2780^Adr^ cells after various treatments using Annexin V-FITC/PI staining. (NS, not significant, **P < 0.01; ***P < 0.001, one-way ANOVA).

Efficient endocytic activity is essential for cellular uptake and intracellular accumulation of therapeutic agents, representing a critical determinant of successful anticancer therapy [25,26]. To validate the CD44-targeting mechanism of HS-Cu@DOX, we first assessed CD44 surface expression in different cell lines using flow cytometry. As shown in Fig. 2A–C, the ovarian cancer cell lines A2780 and ID8 exhibited markedly high CD44 expression, whereas human umbilical vein endothelial cells (HUVEC) showed minimal CD44 expression and served as a low-expression control. This differential expression pattern provided a basis for evaluating the targeting specificity of HS-Cu@DOX. Subsequently, we compared the cellular uptake of HS-Cu@DOX between CD44-overexpressing and low-expressing cells. In A2780^Adr^ and ID8 cells (both CD44-overexpressing), significant time-dependent internalization of HS-Cu@DOX was observed (Fig. 2D, E and Fig. S3A–D, Supporting Information). Notably, substantial intracellular accumulation of HS-Cu@DOX was observed after 6 h, establishing this time point as optimal for subsequent *in vitro* efficacy studies. In contrast, HUVEC cells incubated with HS-Cu@DOX for 6 h showed minimal uptake as examined by fluorescence microscopy, whereas robust internalization was detected in A2780 and ID8 cells, consistent with their differential CD44 expression levels (Fig. S3U, Supporting Information). Furthermore, competitive inhibition assays using excess free hyaluronic acid (HA) significantly reduced cellular accumulation of HS-Cu@DOX in both A2780^Adr^ and ID8 cells, as evidenced by fluorescence microscopy and flow cytometry (Fig. 2F, G and Fig. S3E and F, Supporting Information). These results collectively indicate that cellular internalization of HS-Cu@DOX is predominantly mediated by CD44-dependent active targeting.

To visually the internalization process and provide ultrastructural evidence of nanoparticle uptake, transmission electron microscopy (TEM) was used to directly observe intracellular trafficking events. As shown in Fig. S2, abundant electron-dense HS-Cu@DOX nanoparticles were encapsulated within intracellular membrane-bound vesicular structures in ID8 cells after incubation, whereas no such electron-dense aggregates were observed in untreated control cells. These findings provide direct morphological evidence that HS-Cu@DOX nanoparticles are internalized via an endocytic pathway. Collectively, these results demonstrate that HS-Cu@DOX is efficiently internalized into cancer cells primarily through CD44-mediated active targeting, followed by endocytosis-dependent intracellular delivery.

We next evaluated the *in vitro* cytotoxicity of HS-Cu@DOX in ovarian cancer cell lines (A2780 and ID8) and their drug-resistant counterpart (A2780^Adr^) using the CCK-8 assay. Cells were treated with equivalent doses of DOX, SKN, DOX + SKN, HS-Cu, and HS-Cu@DOX. The results showed that HS-Cu@DOX induced superior cytotoxicity compared with all other treatments in both A2780 and ID8 cells (Fig. 2H and Fig. S3G), likely due to its significantly enhanced cellular uptake efficiency. Critically, HS-Cu@DOX also exhibited potent antitumor activity against chemoresistant A2780^Adr^ cells (Fig. 2I), in contrast to the limited efficacy of free DOX in this resistant cell line (Fig. 2J). Quantitative IC_50_ analysis (Fig. S3J–L, Supporting Information) further substantiated this superior activity. Collectively, these data establish HS-Cu@DOX as a highly effective agent not only against parental ovarian cancer cells but also against chemoresistant phenotypes, highlighting its potential to overcome intrinsic drug resistance. Following determination of IC_50_ values by CCK-8 assay, we performed all subsequent *in vitro* experiments using SKN and DOX at the corresponding concentrations for each cell line. All *in vitro* experiments were performed using SKN and DOX at the indicated concentrations for each cell line (A2780: 0.57 μM SKN, 0.70 μM DOX; ID8: 0.43 μM SKN, 0.52 μM DOX; A2780^Adr^: 1.13 μM SKN, 1.39 μM DOX), with HS-Cu and HS-Cu@DOX administered at equivalent drug doses. Furthermore, to assess the cytotoxicity of HS-Cu@DOX in normal cells, we utilized two human non-cancerous cell lines: immortalized hepatic LO2 cells and renal epithelial HEK293T cells. As shown in Fig. 2K, HS-Cu@DOX exhibited minimal cytotoxicity in these normal cell models. These results indicate that HS-Cu@DOX selectively targets and inhibits tumor cells while inducing negligible adverse effects on normal cellular systems.

Moreover, lactate dehydrogenase (LDH) release assays showed that HS-Cu@DOX induced significant cytotoxicity by compromising plasma membrane integrity, as evidenced by increased LDH release (Fig. 2L and Fig. S3H and I, Supporting Information). Concurrently, EdU assays demonstrated that HS-Cu@DOX effectively suppressed proliferation in both parental and chemoresistant cancer cells (Fig. 2M and Fig. S3M–Q, Supporting Information). Furthermore, colony formation assays (Fig. 2N–P and Fig. S3R–T, Supporting Information), fluorescence images from calcein-AM/propidium iodide (PI) co-staining (Fig. 2Q and Fig. S4A, Supporting Information), and Annexin V/PI flow cytometry (Fig. 2R–T and Fig. S4B and C, Supporting Information) all yielded consistent results with the observed cytotoxicity profiles. Collectively, these multimodal assays demonstrate that HS-Cu@DOX not only exhibits potent antitumor efficacy but also overcomes intrinsic chemoresistance mechanisms in refractory ovarian cancer cells *in vitro*.

### 2.3 HS-Cu@DOX induces cuproptosis through mitochondrial copper overload and metabolic collapse

**Fig. 3:**
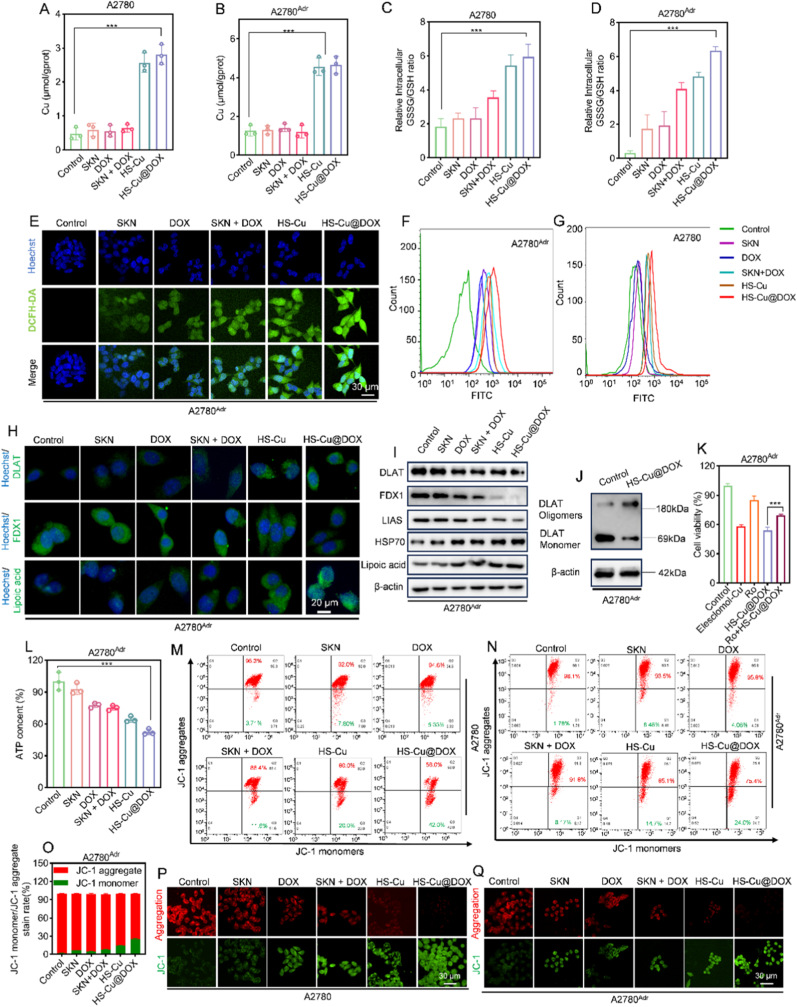
HS-Cu@DOX induced cuproptosis through mitochondrial copper overload and metabolic collapse. SKN and DOX concentrations were set as follows: A2780 (0.57 μM SKN, 0.70 μM DOX), ID8 (0.43 μM SKN, 0.52 μM DOX), and A2780^Adr^ (1.13 μM SKN, 1.39 μM DOX), with HS-Cu and HS-Cu@DOX administered at equivalent drug doses. A, B) Intracellular copper levels in parental and drug-resistant ovarian cancer cells (A2780 and A2780^Adr^ cells) after various treatments. C, D) The ratio of GSSG/GSH in A2780 (C) and A2780^Adr^ (D) cells was measured following different treatments. E, F) Fluorescence pictures and flow cytometry analysis of intracellular ROS level in A2780^Adr^ cells. Scale bar:30 μm. G) Flow cytometry analysis of intracellular ROS level in A2780 cells. H) Fluorescence images of DLAT, FDX1 and Lipoic acid of A2780^Adr^ cells with the indicated treatments. (Green channel, DLAT, FDX1 and Lipoic acid; blue channel, DAPI; scale bar: 20 μm). I) Immunoblotting analysis of DLAT, FDX1, LIAS, HSP70, and lipoic acid in A2780^Adr^ cells treated with different reagents. J) Western blot analysis of cuproptosis-associated DLAT monomers and oligomers in A2780^Adr^ ovarian cancer cells. K) Cell viability was detected in A2780^Adr^ cells with elesclomol (copper inducer) and rotenone (Ro) to verify HS-Cu@DOX-triggered cuproptosis. L) Intracellular ATP secretion levels of A2780^Adr^ cells under various conditions. (n = 3). M − N) Representative flow cytometry of A2780 and A2780^Adr^ cells after JC-1 staining. O) Quantitative analysis of ΔΨm in A2780^Adr^ cells. (n = 3). P-Q) The ΔΨm fluorescence graph in A2780 and A2780^Adr^ cells treated with various treatments (scale bar: 30 μm). (n = 3; ***P < 0.001; one-way ANOVA). (For interpretation of the references to color in this figure legend, the reader is referred to the Web version of this article.)

The mechanism underlying HS-Cu@DOX-induced cuproptosis was investigated in drug-resistant (A2780^Adr^), parental (A2780), and murine (ID8) ovarian cancer models. Initially, HS-Cu@DOX induced significant intracellular copper accumulation in A2780, A2780^Adr^, and ID8 ovarian cancer cells, as evidenced by significantly elevated Cu^2+^ levels compared with the control and other treatment groups (Fig. 3A and B and Fig. S5A, Supporting Information).This copper overload acted as an efficient Fenton-like catalyst, depleting reduced GSH and consequently increasing the glutathione disulfide (GSSG)/GSH ratio (Fig. 3C, D and Fig. S5B, Supporting Information). Subsequently, the elevated intracellular copper levels in tumor cells led to pronounced GSH depletion, triggering Fenton-like reactions and subsequent ROS accumulation (Fig. 3E–G and Fig. S5C–E, Supporting Information), which further induced oxidative stress and mitochondrial damage. Within mitochondria, copper overload promotes direct binding to lipoylated DLAT, resulting in proteotoxic aggregation. FDX1, a master regulator of protein lipoylation, significantly enhances cytotoxic oligomerization of the DLAT complex, ultimately triggering copper-induced cell death [27].

To further investigate HS-Cu@DOX-induced cell death via cuproptosis, we examined key cuproptosis-related markers, including lipoylated DLAT, total DLAT, FDX1, and LIAS. As shown in Fig. 3H and Fig. S5F–G, Supporting Information, immunofluorescence analysis revealed pronounced DLAT aggregation and elevated lipoic acid signals in HS-Cu@DOX-treated A2780^Adr^ cells, accompanied by altered FDX1 expression, indicating copper-dependent disruption of the lipoylation pathway.

Immunoblotting results further confirmed the downregulation of LIAS, DLAT, and FDX1, together with increased HSP70 expression and enhanced lipoic acid–associated lipoylation signals, supporting robust activation of cuproptosis (Fig. 3I and Figure S5H-I, Supporting Information). Consistently, Western blot analysis was performed to directly assess DLAT oligomerization by detecting DLAT monomers and oligomers in A2780^Adr^ and ID8 cells. As shown in Fig. 3J and Fig. S5J, treatment with HS-Cu@DOX markedly increased high-molecular-weight DLAT oligomers, accompanied by a corresponding decrease in DLAT monomer levels compared with the control group. This observation indicates that copper ions delivered by HS-Cu@DOX directly promote aggregation of lipoylated DLAT, a defining hallmark of cuproptosis [28].

Additionally, to functionally validate that HS-Cu@DOX triggers cuproptosis, we assessed its critical role in mediating cell death. As shown in Fig. 3K and Fig. S5K, elesclomol-Cu, a known cuproptosis inducer, significantly reduced A2780^Adr^ cell viability, recapitulating the cuproptotic effect. Importantly, HS-Cu@DOX-induced cytotoxicity was rescued by the copper chelator bathocuproinedisulfonic acid (BCS) and rotenone (Ro), both of which restored cell viability. These findings demonstrate that HS-Cu@DOX-induced cytotoxicity is primarily dependent on cuproptosis.

Next, we assessed intracellular ATP levels in A2780^Adr^, A2780, and ID8 cells. HS-Cu@DOX significantly reduced ATP levels compared with controls (Fig. 3L and Fig. S5L and M, Supporting Information), confirming inhibition of mitochondrial energy production. Consistent with impaired mitochondrial bioenergetics, JC-1 fluorescence imaging and flow cytometry revealed a substantial shift from JC-1 aggregates to monomers in HS-Cu@DOX-treated cells (Fig. 3M–Q and Fig. S6A–D, Supporting Information), demonstrating collapse of mitochondrial membrane potential (ΔΨm) and subsequent mitochondrial dysfunction. Collectively, these data delineate a coherent cuproptosis cascade in which mitochondrial copper overload triggers oxidative stress, leading to lipoylation-dependent DLAT aggregation and FDX1-mediated amplification of oligomerization, ultimately culminating in mitochondrial bioenergetic failure.

### 2.4 HS-Cu@DOX overcomes chemoresistance by eliciting mitochondrial dysfunction and suppressing cancer stemness

Cuproptosis-inducing therapies have demonstrated significant potential for specifically targeting cancer stemness, as documented in recent studies [29]. The reliance of cancer stemness on mitochondrial integrity renders it highly vulnerable to mitochondrial dysfunction [30]. Moreover, results presented in Fig. 3 demonstrate that HS-Cu@DOX effectively triggered cuproptosis, eliciting collapse of mitochondrial membrane potential (ΔΨm) and reduced ATP production. This metabolic disruption potentially contributes to energy depletion, which is critical for sustaining cancer stemness. Given the established dependence of CSC stemness on mitochondrial integrity, we investigated the mechanism by which HS-Cu@DOX targets stemness-related pathways to overcome chemoresistance in ovarian cancer.

**Fig. 4:**
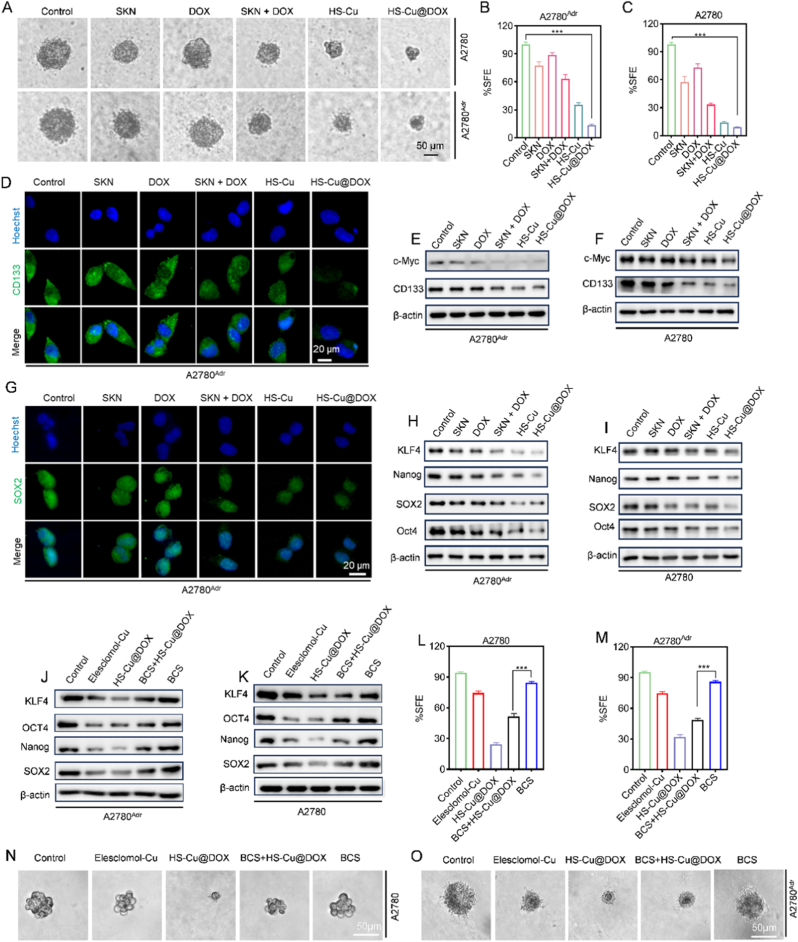
HS-Cu@DOX overcomes chemoresistance by suppressing cancer stemness through cuproptosis-dependent mechanisms. SKN and DOX concentrations were set as follows: A2780 (0.57 μM SKN, 0.70 μM DOX), ID8 (0.43 μM SKN, 0.52 μM DOX), and A2780^Adr^ (1.13 μM SKN, 1.39 μM DOX), with HS-Cu and HS-Cu@DOX administered at equivalent drug doses. A-C) Images and statistical analysis of SFE with SKN, DOX, SKN + DOX, HS-Cu, and HS-Cu@DOX. Scale bar: 50 μm. D) Immunofluorescence analysis of CD133 protein in A2780^Adr^ cells treated with SKN, DOX, SKN + DOX, HS-Cu, and HS-Cu@DOX. Scale bar = 20 μm. E-F) Representative western blotting of CD133 and c-Myc proteins in A2780^Adr^ and A2780 cells. G) Immunofluorescence analysis of SOX2 protein in A2780^Adr^ cells treated with SKN, DOX, SKN + DOX, HS-Cu, and HS-Cu@DOX. Scale bar = 20 μm. H, I) Expression of KLF4, Nanog, SOX2, OCT4, proteins in A2780^Adr^ and A2780 cells using western blotting assay. J-K) Western blot analysis of KLF4, Nanog, SOX2, and OCT4 protein expression in A2780^Adr^ and A2780 cells treated with elesclomol-Cu, BCS, HS-Cu@DOX, and HS-Cu@DOX + BCS. (L-O) Images and statistical analysis of SFE with elesclomol-Cu, BCS, HS-Cu@DOX, and HS-Cu@DOX + BCS. Scale bar: 50 μm. (n = 3; ***P < 0.001; one-way ANOVA).

Since tumorsphere formation represents a functional hallmark of CSCs and sphere formation efficiency (SFE) offers a quantitative measure of stemness [31], we utilized this assay to evaluate the effect of HS-Cu@DOX treatment. As depicted in Fig. 4A–C and Fig. S7A–B (Supporting Information), HS-Cu@DOX-treated groups exhibited significantly reduced tumorsphere volume compared with free DOX controls, indicating effective disruption of CSC propagation potential. Building upon the observed impairment of CSC propagation, we further investigated the molecular mediators of stemness vulnerability. CD133 (Prominin-1) functions as a key CSC surface marker with established correlations to self-renewal capacity and chemoresistance across malignancies [32]. Additionally, c-Myc, a critical oncogene frequently overexpressed in multiple cancer types, including ovarian cancer, plays a key role in amplifying the CD133-dependent stemness program [33,34]. As shown in Fig. 4D and Fig. S7D–E (Supporting Information), membrane localization of CD133 was significantly reduced upon HS-Cu@DOX treatment, supporting the attenuation of the CSC phenotype. Western blot analysis (Fig. 4E–F and Fig. S7C, Supporting Information) revealed that HS-Cu@DOX downregulated protein expression of both CD133 and c-Myc, providing molecular evidence that the stemness-sustaining circuitry is disrupted. In addition, immunofluorescence and immunoblotting analyses confirmed significant downregulation of core stemness-associated transcription factors (KLF4, Nanog, SOX2, OCT4) following HS-Cu@DOX treatment (Fig. 4G–I and Figure S7F-H, Supporting Information).

Importantly, these stemness-associated transcription factors are not merely markers of CSC identity but have been widely reported to function as critical regulators of tumor chemoresistance. Specifically, NANOG, SOX2, OCT4, and KLF family members contribute to chemoresistance by regulating cell cycle progression, promoting epithelial–mesenchymal transition (EMT), reprogramming cellular metabolism toward glycolysis, and suppressing apoptotic signaling. These processes collectively support the establishment and maintenance of a drug-resistant tumor phenotype [7,35,36]. Moreover, NANOG, SOX2, and OCT4 form an interconnected self-sustaining regulatory network that reinforces CSC plasticity and maintains chemoresistant subpopulations [37]. Therefore, the observed downregulation of these transcription factors upon HS-Cu@DOX treatment suggests not only suppression of stemness but also disruption of CSC-driven chemoresistance programs. Collectively, these findings establish that HS-Cu@DOX disrupts the CSC regulatory network, leading to suppression of both stemness maintenance and CSC-associated chemoresistance.

### 2.5 HS-Cu@DOX overcomes chemoresistance by attenuating cancer stemness through cuproptosis induction

To determine whether the suppression of cancer stemness by HS-Cu@DOX is mediated by cuproptosis, we performed pharmacological rescue experiments in chemoresistant A2780^Adr^ and parental A2780 ovarian cancer cells using modulators of copper-dependent cell death. We further examined the protein expression levels of key stemness-associated transcription factors, including KLF4, NANOG, SOX2, and OCT4. As shown in Fig. 4J–K, treatment with the cuproptosis inducer Elesclomol-Cu significantly downregulated the expression of these stemness markers in both A2780^Adr^ and A2780 cells, indicating that cuproptosis may suppress cancer stemness. Consistent with the cell viability results, co-treatment with the copper chelator BCS (HS-Cu@DOX + BCS) effectively reversed the HS-Cu@DOX-induced downregulation of stemness markers, providing direct evidence that HS-Cu@DOX-mediated suppression of cancer stemness is dependent on cuproptosis induction.

Functional validation using tumor sphere formation assays confirmed these findings. Both elesclomol-Cu and HS-Cu@DOX markedly inhibited sphere-forming capacity in both cell lines. Conversely, concomitant treatment with BCS restored the self-renewal capacity impaired by HS-Cu@DOX (Fig. 4L–O), confirming that cuproptosis activation plays a critical role in disrupting cancer stem cell properties. Collectively, these data demonstrate that HS-Cu@DOX exerts potent antitumor effects against chemoresistant ovarian cancer by attenuating cancer stemness via a cuproptosis-dependent mechanism, thereby providing a compelling strategy for reversing chemoresistance.

### 2.6 *In Vivo* Biodistribution and Antitumor Efficacy of HS-Cu@DOX in parental A2780 Xenografts

**Fig. 5:**
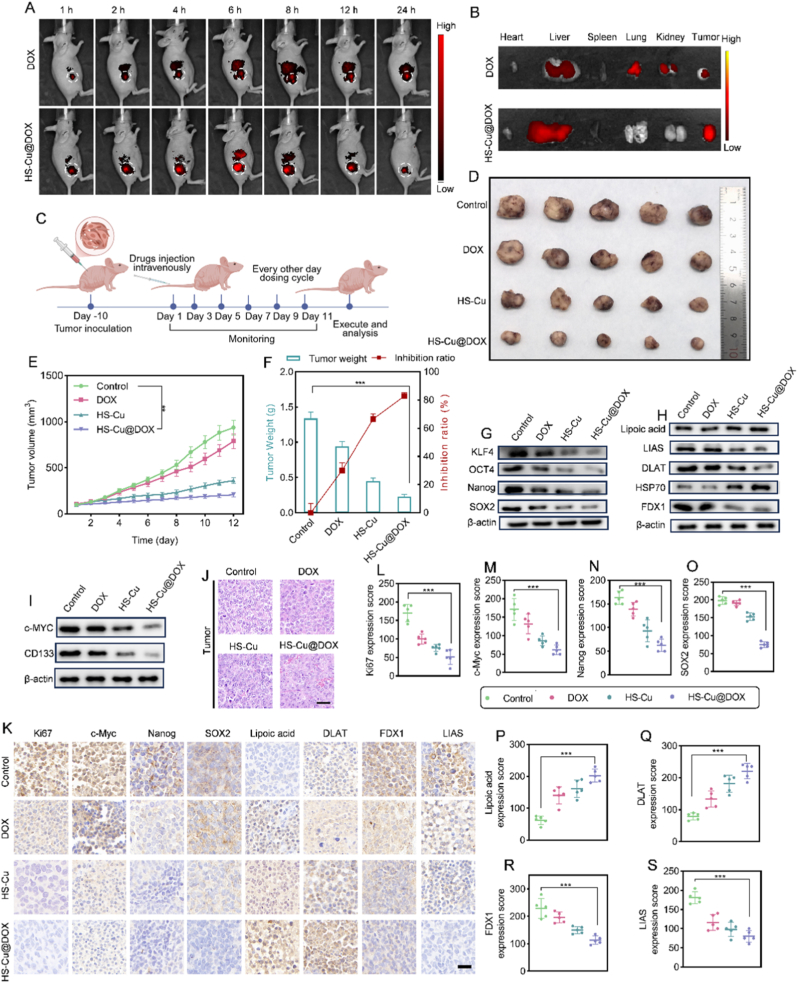
*In Vivo* Biodistribution and Antitumor Efficacy of HS-Cu@DOX in Parental A2780 Xenografts. A) Real-time *in vivo* imaging of DOX and HS-Cu@DOX following intravenous injection in the A2780 tumor-bearing BALB/c nude mice model. B) *Ex vivo* organ and tumor luminescence at 24 h post-dose. C) Schematic of the A2780 tumor therapy regimen in BALB/c nude mice. Created with BioRender.com. D) Images depicting individual tumors (n = 5). E) Tumor volume by group at designated time points (n = 5; **P < 0.01; one-way ANOVA). F) Tumor weight and inhibition rate by group (n = 5; ***P < 0.001; one-way ANOVA). G) Expression of KLF4, Nanog, SOX2, OCT4, proteins in tumor tissues following various strategies. H) Expression of DLAT, FDX1, LIAS, HSP70, and lipoic acid proteins in tumor tissues following various strategies. I) Expression of CD133 and c-Myc proteins in tumor tissues following various strategies. J) H&E staining of tumor tissue from all groups. Scale bar = 50 μm. K-S) Immunohistochemical staining and corresponding quantitative expression scores of tumor tissues for various markers after treatment with different formulations. Scale bar = 50 μm. (***P < 0.001; one-way ANOVA).

The *in vivo* fluorescence imaging system was employed to monitor the fluorescence distribution of DOX and HS-Cu@DOX following intravenous administration via tail vein injection in subcutaneous tumor-bearing BALB/c mice at various time points. As shown in Fig. 5A, HS-Cu@DOX exhibited superior tumor accumulation compared with the DOX group, as evidenced by significantly higher fluorescence intensity at the tumor site. This finding indicated enhanced tumor-targeting capability attributed to HA modification. Twenty-four hours post-injection, *ex vivo* fluorescence imaging of major organs and tumor tissues revealed sustained fluorescence retention of HS-Cu@DOX in the tumor site (Fig. 5B). Collectively, these results demonstrate that HS-Cu@DOX enables efficient tumor targeting, thereby enhancing drug accumulation at the tumor site and improving therapeutic efficacy.

To evaluate the therapeutic efficacy of HS-Cu@DOX *in vivo*, an A2780 subcutaneous xenograft model was established. Tumor-bearing mice were randomly divided into four groups receiving saline, DOX, HS-Cu, or HS-Cu@DOX (Fig. 5C). Starting 10 days after A2780 cell inoculation, mice were administered saline, DOX, HS-Cu, or HS-Cu@DOX via intravenous (tail vein) injection every other day for 13 days (six injections in total). Tumor size was monitored daily, and tumor volume was calculated accordingly. At the end of the treatment period, mice were euthanized, and tumors were excised, weighed, and photographed. Notably, as illustrated in Fig. 5D–F, all treatment groups exhibited varying degrees of tumor growth inhibition, with HS-Cu@DOX showing the most pronounced reduction.

Subsequently, Western blot analysis of excised tumors (Fig. 5G–I) revealed that the expression levels of cuproptosis-related proteins, stemness regulators, and stemness markers were consistent with the trends observed in our *in vitro* findings. Hematoxylin and eosin (H&E) staining further demonstrated extensive tumor cell necrosis and structural disruption in the HS-Cu@DOX group (Fig. 5J). Immunohistochemical (IHC) analysis of tumor tissues (Fig. 5K–S) revealed distinct expression patterns across treatment groups. Specifically, staining intensities for the proliferation marker Ki-67 and the stemness-associated markers (c-Myc, Nanog, SOX2) were markedly reduced in the HS-Cu@DOX group compared with other treatments. Concurrently, IHC assessment of key cuproptosis-related markers, namely lipoic acid, DLAT, FDX1, and LIAS, revealed significant upregulation of lipoic acid and DLAT and pronounced downregulation of FDX1 and LIAS, exclusively in HS-Cu@DOX-treated tumors (Fig. 5K–S), collectively indicating robust induction of cuproptosis. Together, these findings demonstrate that HS-Cu@DOX effectively suppresses tumor growth *in vivo* by triggering cuproptosis and attenuating cancer stemness within tumor tissues.

To further address concerns regarding long-term therapeutic durability and potential tumor regrowth after treatment cessation, additional extended *in vivo* experiments were conducted. In this supplementary study, A2780 tumor-bearing BALB/c nude mice were treated when tumor volumes reached approximately 80 mm^3^ (Fig. S8A). The mice received intravenous administrations every other day for a total of eight doses, after which treatment was discontinued. Tumor growth was subsequently monitored until day 20, at which point all animals were sacrificed and tumors were harvested for further analysis, enabling evaluation of both the treatment phase and the post-treatment (drug-withdrawal) phase.

As shown in Fig. S8B, HS-Cu@DOX-treated mice exhibited significant tumor growth inhibition during the treatment period compared with the control group. Although a mild increase in tumor volume was observed following drug withdrawal, the growth rate remained markedly slower than that of the control group, indicating that HS-Cu@DOX exerts not only a potent inhibitory effect during administration but also a sustained suppressive effect on tumor regrowth within the observation window. At the endpoint, tumor weight measurements and tumor inhibition rate analysis further confirmed the superior antitumor efficacy of HS-Cu@DOX (Fig. S8C, ***P < 0.001), consistent with tumor volume trends and representative images of excised tumors (Fig. S8D). Importantly, no significant body weight loss was observed in any treatment group throughout the study period (Fig. S8E), suggesting that HS-Cu@DOX does not induce overt systemic toxicity and is well tolerated *in vivo.*

### 2.7 HS-Cu@DOX Overcomes Chemoresistance and Suppresses Tumor Growth in A2780^Adr^ Xenograft Models *In Vivo*

**Fig. 6:**
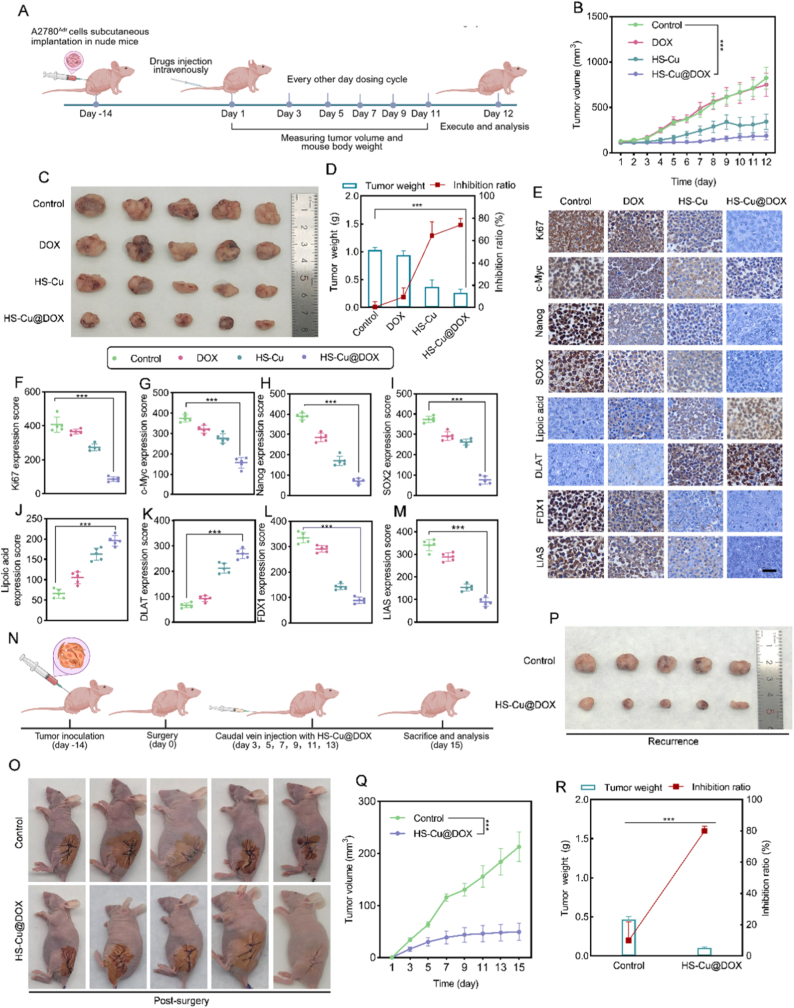
HS-Cu@DOX Overcomes Chemoresistance and Suppresses Tumor Growth in A2780^Adr^ Xenograft Models *In Vivo.* A) The schematic illustrates the treatment regimen establishment and administration in the A2780^Adr^ mouse model. Created with BioRender.com. B) Tumor volume by group at designated time points (n = 5; ***P < 0.001; one-way ANOVA). C) Images depicting individual tumors (n = 5). D) Tumor weight and inhibition rate by group (n = 5; ***P < 0.001; one-way ANOVA). E-M) Immunohistochemical staining and corresponding quantitative expression scores of tumor tissues for various markers after treatment with different formulations. Scale bar = 50 μm. (***P < 0.001; one-way ANOVA). N) Schematic of the tumor recurrence model and the administered treatments. Created with BioRender.com. O) Corresponding images of tumor excision (n = 5). P) Tumor gross morphology across experimental groups (n = 5). Q) Tumor volume by group at designated time points (n = 5; ***P < 0.001; one-way ANOVA). R) Tumor weight and inhibition rate by group (n = 5; ***P < 0.001; one-way ANOVA).

To further evaluate the ability of HS-Cu@DOX to overcome chemoresistance *in vivo*, and based on its confirmed tumor-targeting and antitumor efficacy (Fig. 5), an A2780^Adr^ xenograft model in BALB/c nude mice was established (Fig. 6A). After inoculation of A2780^Adr^ cells, mice were treated with saline, DOX, HS-Cu, or HS-Cu@DOX via tail vein injection every other day for 12 days. Throughout the experiment, body weight, tumor size, and tumor volume were monitored. At the end of the treatment period, mice were euthanized, and tumors were excised for subsequent measurement of volume and weight. Consistent with the results obtained in the parental A2780 model (Section 2.6), HS-Cu@DOX exhibited the most potent tumor growth inhibition in the resistant A2780^Adr^ model, as evidenced by significantly reduced tumor volume and weight (Fig. 6B–D). This effect was accompanied by markedly decreased expression of proliferation and stemness markers, as well as pronounced alterations in cuproptosis-related proteins (Fig. 6E–M).

Additionally, to evaluate the ability of HS-Cu@DOX to inhibit tumor recurrence, a tumor recurrence model was established as illustrated in Fig. 6N. Complete tumor resection was visually confirmed by postoperative surgical site photographs (Fig. 6O). During the 15-day experimental period, mice received equal doses of the corresponding formulations via intravenous injection every two days. As shown in Fig. 6P–R, compared with the control group, HS-Cu@DOX treatment significantly suppressed tumor recurrence. In conclusion, the developed HS-Cu@DOX system exhibited comprehensive antitumor efficacy, including inhibition of tumor growth, reversal of chemoresistance, and reduction of tumor recurrence risk, without obvious systemic toxicity.

### 2.8 Assessment of HS-Cu@DOX biosafety

**Fig. 7:**
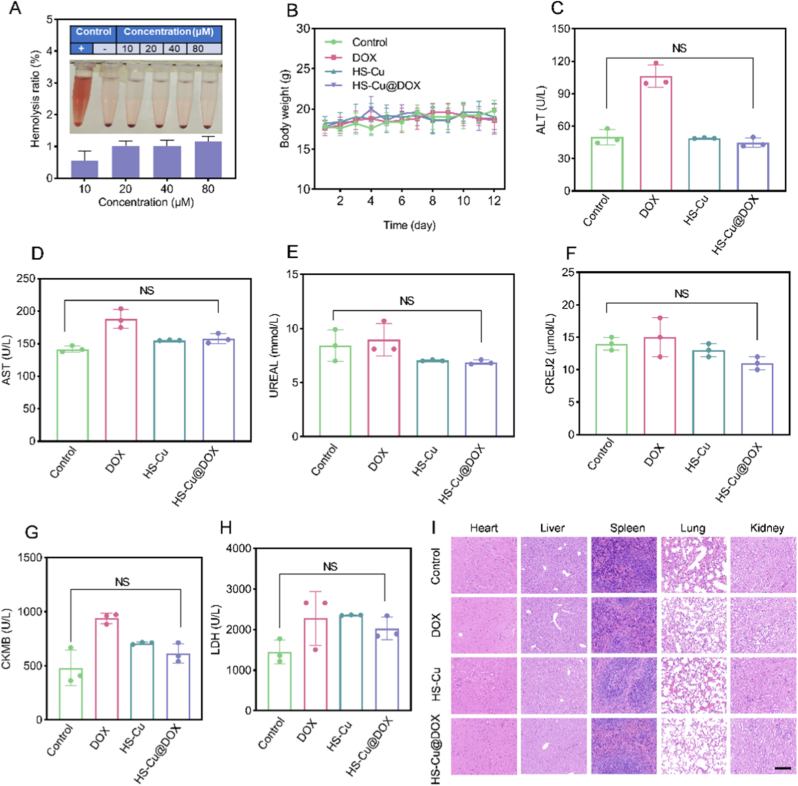
Assessment of HS-Cu@DOX Biosafety. A) Hemolysis rate and photographs of HS-Cu@DOX at different concentrations. B) Body weight of mice in different groups (n = 5). C-H) Analysis of the serum biochemistry indicators (ALT (C); AST (D); UREAL (E) CREJ2 (F); CKMB (G), LDH (H)) after treatment with saline, DOX, HS-Cu, and HS-Cu@DOX (n = 3, one-way ANOVA, NS, not significant). I) Representative H&E images of major organs across experimental groups (Scale bars: 50 μm).

To investigate the hemocompatibility of HS-Cu@DOX prior to intravenous injection, a hemolysis assay was performed (Fig. 7A). The hemolysis ratios of all samples remained below 5% across the tested concentration range, indicating good blood compatibility of HS-Cu@DOX nanoparticles suitable for intravenous use [38].

Furthermore, body weight changes in mice were monitored throughout the *in vivo* treatment period. No significant differences were observed among the treatment groups and the saline control group (Fig. 7B), indicating the absence of systemic toxicity. Moreover, to comprehensively assess organ function, key serum biochemical markers were quantified, including alanine aminotransferase (ALT) and aspartate aminotransferase (AST) for hepatic function, blood urea nitrogen (UREA) and creatinine (CRE) for renal function, and creatine kinase-MB (CK-MB) and lactate dehydrogenase (LDH) as indicators of myocardial injury. These parameters are widely used indicators of liver, kidney, and cardiac function [39]. As shown in Fig. 7C–H, all biochemical indices in the HS-Cu@DOX group remained within normal ranges and showed no significant differences compared with the control group, suggesting no detectable impairment of major organ function.

Finally, histopathological analysis of major organs (heart, liver, spleen, lungs, and kidneys) by H&E staining (Fig. 7I) revealed well-preserved tissue architecture with no observable pathological abnormalities in the HS-Cu@DOX group, further confirming its favorable *in vivo* biosafety profile at the histological level.

## 3 Discussion

Drug resistance remains a major obstacle in ovarian cancer treatment, while cell stemness is a key contributor to chemoresistance and tumor recurrence [40]. In this study, we developed a hyaluronic acid (HA)-functionalized shikonin–copper nanoplatform (HS-Cu@DOX) integrating cuproptosis induction with stemness suppression, achieving potent antitumor efficacy against chemoresistant ovarian cancer.

HS-Cu@DOX was constructed via coordination between shikonin and Cu^2+^, forming a stable nanostructure (∼172 nm) suitable for EPR-mediated tumor accumulation. HA modification enhanced colloidal stability and enabled CD44-targeted delivery, while zeta potential changes confirmed successful DOX loading. Mechanistically, HS-Cu@DOX induced cuproptosis via mitochondrial copper accumulation, resulting in GSH depletion, ROS overproduction, DLAT aggregation, and FDX1 downregulation, leading to mitochondrial dysfunction. Concurrently, it suppressed cancer stemness, as shown by decreased CD133, c-Myc, and pluripotency factors (KLF4, NANOG, SOX2, OCT4), along with impaired tumorsphere formation; these effects were reversed by BCS, confirming cuproptosis involvement. *In vivo*, it significantly inhibited tumor growth, reduced proliferation and stemness markers, and activated cuproptosis pathways, while also suppressing tumor regrowth after treatment withdrawal, indicating durable efficacy. Biosafety evaluations (hemolysis, serum biochemistry, and histopathology) confirmed a favorable safety profile.

Although the use of copper-based agents raises concerns regarding systemic metal homeostasis, HA modification exploits the overexpression of CD44 in ovarian cancer to achieve selective tumor accumulation, thereby reducing off-target copper exposure [11,41]. In addition, the GSH-responsive release behavior of HS-Cu@DOX enables tumor-specific co-delivery of the cuproptosis inducer and chemotherapeutic drug, enhancing synergistic effects within the tumor microenvironment. This design not only improves the eradication of drug-resistant cells but also helps reduce systemic toxicity and expand the therapeutic window. However, several challenges remain before clinical translation can be realized, including the limited translatability of the EPR effect in human tumors, the need for comprehensive evaluation of long-term copper metabolism and potential accumulation, as well as further validation of large-scale reproducibility, formulation stability, and pharmacokinetics in higher animal models. Addressing these issues will be essential to bridge the gap between preclinical efficacy and clinical application. Overall, HS-Cu@DOX represents a promising nanotherapeutic strategy by integrating cuproptosis induction with cancer stemness targeting, offering a synergistic approach for overcoming chemoresistance in ovarian cancer and supporting further development of metal-based nanomedicines toward clinical translation.

## 4 Conclusion

In summary, we successfully developed a self-assembly–driven nanoplatform, HS-Cu@DOX, which co-delivers shikonin and DOX to overcome chemoresistance in ovarian cancer. HS-Cu@DOX effectively addresses the limitations of shikonin by improving its dispersibility and bioavailability through copper coordination and nanoformulation. The released SKN-Cu complex not only depletes GSH and induces cuproptosis but also potently suppresses cancer stemness by downregulating key transcription factors, thereby disrupting the self-renewal capacity of cancer stem cells (CSCs). Importantly, HS-Cu@DOX demonstrated enhanced therapeutic efficacy in both *in vitro* and *in vivo* models, effectively reversing DOX resistance and delaying tumor recurrence. Overall, this study provides a novel therapeutic strategy that simultaneously targets chemoresistant cells through Cu-induced cell death and stemness pathway inhibition, offering a promising approach for the treatment of recurrent ovarian cancer.

## 5 Experimental section

### 5.1 Materials

Hyaluronic acid (cat. H131007); purity ≥99%. Fetal bovine serum (FBS) and Dulbecco's modified Eagle medium (DMEM) were purchased from Thermo Fisher Scientific. Detailed product codes and suppliers for all commercially sourced antibodies are provided in Table S1. For immunoblotting, immunofluorescence, and immunohistochemistry assays, antibodies were used at dilution ratios of 1:1,000, 1:200, and 1:200, respectively. Product codes and suppliers for all commercial assay kits and chemical reagents are listed in Tables S2 and S3.

### 5.2 Preparation of HS-Cu@DOX

Shikonin (SKN) was dissolved in 3 mL of an ethanol–aqueous mixed solvent (ethanol: ddH_2_O = 3:1, v/v) under sonication until complete dissolution. Copper (II) nitrate trihydrate (Cu(NO_3_)_2_·3H_2_O) was dissolved in 1 mL of ddH_2_O. For coordination, the copper solution was added dropwise to the SKN solution at a molar ratio of SKN:Cu^2+^ = 1:1 under magnetic stirring at 800 rpm, and the reaction was allowed to proceed for 6 h at room temperature. The resulting shikonin–copper coordination complex (denoted as S-Cu) was collected by centrifugation at 12,000 rpm for 10 min, washed three times with ethanol and ddH_2_O, redissolved in water, and lyophilized to obtain powdered S-Cu.

Next, 1 mg of S-Cu was dispersed in 1 mL of water by sonication for 5 min. Hyaluronic acid (HA, 2 mg in 0.5 mL water) was added dropwise under stirring. The pH was adjusted to 8–9, and the mixture was stirred for 24 h to obtain HA-modified nanoparticles (HS-Cu), which were purified by centrifugation and washing as described above.

Finally, DOX loading was achieved via electrostatic interactions between the carboxyl groups of HA and the protonated amino groups of DOX. Briefly, 1 mg of lyophilized HS-Cu was dispersed in 2 mL of ddH_2_O by sonication. DOX·HCl was dissolved in water and added dropwise under light-protected conditions with stirring at 800 rpm for 24 h. The resulting HS-Cu@DOX nanoparticles were collected by centrifugation (12,000 rpm, 10 min), washed three times with ddH_2_O until the supernatant became nearly colorless, resuspended, and lyophilized. The final product was stored at 4 °C in the dark.

### 5.3 Characterization of HS-Cu@DOX

The zeta potential and size distribution of HS-Cu and HS-Cu@DOX were measured using a nanoparticle tracking analyzer (ZetaView PTM, ParticleMetrix). Their morphology was examined by transmission electron microscopy (HT7800, Hitachi). The morphology and elemental composition of S-Cu were characterized using scanning electron microscopy (Sigma 360, ZEISS, Germany), and its chemical states were analyzed by X-ray photoelectron spectroscopy (XPS, K-Alpha, Thermo Scientific). HS-Cu@DOX, HS-Cu, SKN, and S-Cu were characterized using a UV-3600 UV–Vis spectrophotometer (Shimadzu, Japan) and a Nicolet iS10 FTIR spectrometer (Thermo Fisher Scientific). Additionally, fluorescence emission of HS-Cu@DOX was measured using a multimode microplate reader. All these measurements were performed at the West China School of Basic Medical Sciences & Forensic Medicine, Sichuan University.

### 5.4 Drug loading efficiency of HS-Cu@DOX

The drug loading content (DLC) and encapsulation efficiency (EE) of SKN and DOX in HS-Cu@DOX were calculated using the following equations. The concentrations of SKN and DOX in the nanoparticles were determined by UV–Vis spectroscopy and high-performance liquid chromatography (HPLC, LC-2030C Plus, Shimadzu), respectively, with the aid of their respective standard curves.

$$\text{The}\;\text{drug}\;\text{loading}\;\text{of}\;\text{SKN}\;\left(\%\right)=\frac{C_{SKN}\times V}{C_{SKN}\times V+M_{HA}}\times 100\%$$

$$\text{The}\;\text{drug}\;\text{loading}\;\text{of}\;\text{DOX}\;\left(\%\right)=\frac{C_{\text{DOX}}\times V}{C_{SKN}\times V+C_{\text{DOX}}\times V+M_{HA}}\times 100\%$$

where *C*_*SKN*_ and *C*_*DOX*_ denoted the concentrations of SKN and DOX in HS-Cu@DOX, respectively, *V* represented the solution volume, and M_*HA*_ is the mass of HA.

The Cu^2+^ content in HS-Cu@DOX nanoparticles was quantified using inductively coupled plasma optical emission spectrometry (ICP-OES). Briefly, a known amount of freeze-dried HS-Cu@DOX nanoparticles (m_0_, g) was accurately weighed and completely digested in an appropriate volume (V_0_, mL) of nitric acid solution to release all encapsulated Cu^2+^. The resulting solution was diluted to a suitable concentration and measured by ICP-OES to obtain the Cu^2+^ concentration (C_1_, mg/L). The loading efficiency (W, %) of Cu^2+^ in the nanoparticles was calculated according to the following equations:

$$C_{x}=\frac{C_{0}fV_{0}}{m_{0}}$$

$$W\left(\%\right)=\frac{C_{x}}{10^{6}}\times 100\%$$

where m_0_ represented the mass of the sample taken for analysis (g), as recorded by an analytical balance;

V0 denoted the volume of the solution after sample digestion and dilution to a fixed volume (mL);

f was the dilution factor;

C_0_ was the concentration of the element in the test solution (mg/L or μg/L), as measured by the instrument;

Cx represented the final concentration of the element in the sample (mg/kg or μg/kg); and W (%) indicated the final concentration expressed as a percentage.

### 5.5 *In vitro* release of SKN, DOX, and Cu from HS-Cu@DOX

The release profiles of SKN and DOX from HS-Cu@DOX were investigated using a dialysis method. Briefly, 1 mL of HS-Cu@DOX solution (2 mg/mL) was placed into dialysis bags (MWCO = 3500 Da). After sealing, the bags were immersed in 25 mL of PBS release medium under two conditions: pH 7.4 and pH 7.4 supplemented with 10 mM glutathione (GSH). The release experiments were conducted at 37 °C under constant shaking. At predetermined time intervals, 8 mL of release medium was withdrawn and replaced with an equal volume of fresh PBS buffer. The concentrations of SKN and DOX were determined using their respective standard curves. In parallel, the release of copper (Cu) from HS-Cu@DOX was evaluated under the same conditions and Cu concentrations in the release medium at each time point was quantified by inductively coupled plasma mass spectrometry (ICP-MS) at the Analytical and Testing Center of Sichuan University.

### 5.6 Determination of GSH depletion by HS-Cu@DOX via DTNB assay

The responsiveness of HS-Cu@DOX to glutathione (GSH) was systematically evaluated using the 5,5′-dithiobis-(2-nitrobenzoic acid) (DTNB) method. The procedure was conducted as follows: first, 120 μL of DTNB in phosphate buffer (2.5 mg/mL), HS-Cu@DOX (200 μg/mL), and 15 μL of an aqueous GSH solution (10 mM) were added to varying volumes of deionized water to achieve final HS-Cu@DOX concentrations of 0, 5, 10, 20, and 40 μg/mL. After thorough mixing, the samples were incubated at 25 °C for 30 min under constant temperature, followed by centrifugation to remove HS-Cu@DOX. The absorbance of the supernatant was then measured at 412 nm using UV–Vis spectroscopy to quantify assess GSH consumption. All experiments were performed in triplicate to ensure reliability.

### 5.7 Characterization of hydroxyl radical (·OH) generation capacity of HS-Cu@DOX using methylene blue (MB)

A stock solution of HS-Cu@DOX (1 mg/mL) was diluted with deionized water to obtain a working concentration of 200 μg/mL. Then, 1 mL of the diluted solution was mixed with 1 mL of glutathione (GSH, 10 mM) and incubated for 30 min. A parallel control was prepared under identical conditions in the absence of GSH. After incubation, methylene blue (MB) and hydrogen peroxide (H_2_O_2_) were added to each mixture to final concentrations of 10 μg/mL and 110 mM, respectively. The absorbance changes were monitored by UV-Vis spectroscopy in three groups: MB alone, HS-Cu@DOX without GSH, and HS-Cu@DOX with 10 mM GSH. All measurements were performed in triplicate.

### 5.8 Hemolysis assay

Blood samples were collected from the hearts of New Zealand rabbits and centrifuged, followed by repeated washing to obtain erythrocyte pellets. The erythrocyte pellets were then resuspended in normal saline to prepare a 2% (v/v) red blood cell (RBC) suspension. The study included three experimental groups, each performed in triplicate: the experimental group, in which the RBC suspension was mixed at a 1:1 ratio with varying concentrations of HS-Cu@DOX nanoparticles; the negative control group, in which the RBC suspension was mixed with an equal volume of normal saline; and the positive control group, in which the RBC suspension was mixed 1:1 with double-distilled water. All samples were incubated at 37 °C for 3 h in a water bath and then centrifuged at 1000 rpm for 15 min. The absorbance of the supernatant from each group was measured at 540 nm using a UV–Vis spectrophotometer. The hemolysis rate was calculated using the following formula:

$$\text{Hemolysis}\;\text{rate}\left(\%\right)=\frac{A_{S}-A_{N}}{A_{P}-A_{N}}\times 100\%$$

where A_S_, A_N_, and A_P_ represent the absorbance values of the test, negative control, and positive control groups, respectively.

### 5.9 Cell culture

The human ovarian cancer cell lines, including the doxorubicin-resistant variant A2780^Adr^ and its parental line A2780, as well as the murine ovarian cancer cell line ID8, were obtained from the American Type Culture Collection (ATCC; Manassas, VA, USA). All cell lines were cultured in DMEM supplemented with 10% fetal bovine serum (FBS; HyClone, SH30088.03), 100 U/mL penicillin, and streptomycin (Invitrogen). Cells were maintained in a humidified incubator at 37 °C with 5% CO_2_.

### 5.10 Cellular uptake assay

Cellular uptake of HS-Cu@DOX at various time points (0h, 1h, 2h, 4h, 6h, 8h) was evaluated using a fluorescence imaging system (Nikon, Tokyo, Japan) and flow cytometer (BD, FACS Celesta, West China School of Basic Medical Sciences & Forensic Medicine, Sichuan University), respectively.

To assess CD44 surface expression, HUVEC, A2780, and ID8 cells were stained with FITC-conjugated anti-CD44 antibody or isotype control (ID8 + lsotype FITC; ID8 + anti-CD44-FITC; A2780 + lsotype FITC; A2780 + anti-CD44-FITC; HUVEC + lsotype FITC; HUVEC + anti-CD44-FITC) and analyzed by flow cytometry. For comparative uptake, the three cell lines were incubated with HS-Cu@DOX for 6 h and imaged (scale bar: 30 μm). Competitive inhibition assays were performed in A2780Adr and ID8 cells pretreated with excess free HA (2 mg/mL, 30 min), followed by HS-Cu@DOX for 6 h. Uptake was examined by fluorescence microscopy and flow cytometry.

### 5.11 Transmission electron microscopy (TEM) observation of nanoparticle internalization by cells

Cell pellets (approximately 1 × 10^7^ cells) were immediately fixed in 2.5% glutaraldehyde at 4 °C for 12-24 h. After fixation, the samples were rinsed three times with PBS (pH 7.4) for 15 min each, post-fixed with 1% tetroxide (OsO_4_) for 1-2 h, and then washed again three times with 0.1 M PBS (pH 7.4) for 15 min each. Dehydration was performed at room temperature using a graded acetone series (30%, 50%, 70%, 80%, 95%, and 100%), with each step lasting 15 min, followed by two changes of 100% acetone for 20 min each. The samples were infiltrated with a graded series of acetone and EMBed 812 resin at 37 °C: 3:1 for 1 h, 1:1 for 3 h, and pure EMBed 812 overnight. After infiltration, the samples were embedded in pure EMBed 812 and polymerized at a 60 °C oven for at least 48 h. The resin blocks were trimmed and sectioned into 70–90 nm ultrathin slices using an ultramicrotome. The sections were collected onto copper grids, stained with uranyl acetate for 8–15 min followed by lead citrate for 5–10 min, and observed using a transmission electron microscope (80 kV).

### 5.12 CCK-8 assay

A2780^Adr^, A2780, and ID8 cells were seeded in 96-well plates at a density of 3 × 10^3^ cells per well and allowed to adhere overnight. Subsequently, the cells were treated with varying concentrations of SKN, DOX, KN + DOX, HS-Cu, and HS-Cu@DOX for 6 h. After treatment, the drug-containing medium was removed, and cells were cultured in fresh medium for an additional 18 h. At the end of the incubation period, 10 μL of CCK-8 reagent was added to each well, including background control wells. The plate was then returned to a 37 °C incubator and incubated for 3 h. The absorbance at 450 nm was measured using a microplate reader.

### 5.13 Colony formation assay

The long-term antiproliferative effects were assessed by a colony formation assay. Briefly, cells were seeded in 24-well plates at a density of 5 × 10^2^ cells per well and cultured for 72 h. Subsequently, cells were treated with SKN, DOX, SKN + DOX, HS-Cu, or HS-Cu@DOX for 6 h. After treatment, the medium was removed and cells were washed with PBS. The medium was replaced every 3 days. After 10 days of incubation, colonies were fixed and stained with crystal violet for 30 min, followed by three washes with ultrapure water. Images of stained colonies were captured for analysis.

### 5.14 EdU assay

Cells were seeded in 96-well plates and treated with drugs according to the protocol described for the CCK-8 assay. Cell proliferation was assessed using a commercial EdU assay kit (RIBOBIO, C10310-1).

### 5.15 LDH assay

Cells were seeded in 96-well plates at a density of 8 × 10^3^ cells per well and treated under the designated experimental conditions. Subsequently, the supernatant from each well was incubated with LDH reagent at 37 °C for 30 min in the dark. Absorbance was measured to determine LDH activity.

### 5.16 Detection of intracellular ROS

Intracellular ROS levels were measured using a DCFH-DA assay kit. A2780^Adr^, A2780, and ID8 cells were seeded in 6-well or 24-well plates and cultured for 24 h. Subsequently, cells were treated with SKN, DOX, SKN + DOX, HS-Cu, or HS-Cu@DOX. Prior to treatment, cells were incubated with 10 μM DCFH-DA at 37 °C for 20 min according to the manufacturer's instructions. After two washes with PBS, intracellular ROS levels were qualitatively and quantitatively analyzed using inverted fluorescence microscopy and flow cytometry.

### 5.17 Apoptosis assay

Briefly, cells were seeded in 6-well plates at a density of 5 × 10^4^ cells per well and cultured for 24 h. Subsequently, cells were treated with SKN, DOX, SKN + DOX, HS-Cu, or HS-Cu@DOX for 6 h, followed by incubation for an additional 18 h. Cells were then co-stained with Annexin V-FITC and propidium iodide (PI) for 15 min at 4 °C in the dark. Apoptosis was analyzed using flow cytometry according to the manufacturer's instructions.

### 5.18 ATP release assay

Intracellular ATP levels were measured using an ATP Assay Kit. Cells were cultured in 6-well plates for 24 h. Afterward, cells were lysed, and the lysates were centrifuged at 12,000 × g for 5 min at 4 °C to collect the supernatant. According to the manufacturer's instructions, ATP content in the supernatant was determined by measuring luminescence intensity using a 96-well microplate reader.

### 5.19 Assessment of mitochondrial membrane potential

Changes in mitochondrial membrane potential (ΔΨm) were measured using the JC-1 assay. Cells were seeded in 6-well plates at a density of 5 × 10^4^ cells per well and cultured overnight at 37 °C. Subsequently, the cells were treated with SKN, DOX, SKN + DOX, HS-Cu, or HS-Cu@DOX, with normal saline used as a control. Following the treatment, ΔΨm was evaluated according to the manufacturer's instructions using both flow cytometry and fluorescence microscopy.

### 5.20 Immunoblotting

Cells were seeded in 6-well plates at a density of 5 × 10^4^ cells per well and cultured for 24 h. After treatment, cells were harvested, washed with ice-cold PBS, and lysed in radioimmunoprecipitation assay (RIPA) buffer with sonication. Protein samples were separated by sodium dodecyl sulfate–polyacrylamide gel electrophoresis (SDS–PAGE) and transferred onto polyvinylidene difluoride (PVDF) membranes. Membranes were blocked with 5% skim milk for 2 h at room temperature and incubated with primary antibodies overnight at 4 °C with gentle shaking. Subsequently, the membranes were incubated with corresponding secondary antibodies for 2 h. Protein bands were visualized using a ChemiScope 6000 Touch imaging system (Clinx, Shanghai) after incubation with Immobilon Western HRP substrate (Millipore, WBKLS0050).

### 5.21 Immunofluorescence assay

Cells were cultured on sterilized coverslips in 24-well plates for 24 h. After treatment, cells were fixed with 4% paraformaldehyde for 30 min, permeabilized with 0.4% Triton X-100, and blocked with 5% BSA for 1.5 h. Samples were incubated with primary antibodies at 4 °C overnight (∼15 h), followed by incubation with Alexa Fluor-conjugated secondary antibodies for 2 h at room temperature. Cell nuclei were stained with DAPI for 8 min, followed by thorough washing with PBS. Images were acquired using a confocal laser scanning microscope (Nikon, Japan).

### 5.22 Determination of copper concentration

Cells were seeded in 6-well plates at a density of 5 × 10^5^ cells per well and incubated overnight at 37 °C. Following treatments (normal saline, SKN, DOX, SKN + DOX, HS-Cu, or HS-Cu@DOX; n = 3), cells were harvested and processed according to the manufacturer's instructions of the Copper (Cu^2+^) Colorimetric Assay Kit.

### 5.23 Animals models

Female BALB/c nude mice (6 weeks old, weighing 16-22 g) were obtained from Beijing Vital River Laboratory Animal Technology Co., Ltd. All animal experiments were conducted in accordance with the ARRIVE guidelines and the NIH Guide for the Care and Use of Laboratory Animals and were approved by the Animal Ethics Committee of Sichuan University. Tumor xenograft models were established by subcutaneous injection of A2780 or A2780^Adr^ cells into the flank region of each mouse. When tumor volumes reached 80-100 mm^3^, the mice were randomly assigned to four groups for subsequent experimental treatments.

### 5.24 *In Vivo* Biodistribution of HS-Cu@DOX

When subcutaneous tumor volumes reached approximately 80 mm^3^, tumor-bearing BALB/c mice were intravenously injected with free DOX or HS-Cu@DOX via the tail vein at an equivalent DOX dose of 2 mg/kg. Fluorescence imaging and biodistribution analyses were performed at predetermined time intervals (1, 2, 4, 6, 8, 12, and 24 h) using an *in vivo* imaging system. At 24 h post-injection, the mice were euthanized, and major organs were collected for *ex vivo* fluorescence imaging.

### 5.25 Evaluating the antitumor efficacy of HS-Cu@DOX

Subcutaneous tumor models were established by subcutaneous injection of A2780 or A2780^Adr^ cells as previously described. Once tumor volumes reached 80–100 mm^3^, tumor-bearing mice were randomly assigned to four groups (n = 5) and intravenously injected with normal saline, DOX, HS-Cu, or HS-Cu@DOX (2 mg/kg in 100 μL) every two days. Tumor size and body weight were measured daily. On day 12, all mice were euthanized, and tumors and major organs were harvested for further analysis, including hematoxylin-eosin (H&E) staining and immunohistochemistry. Blood samples collected via retro-orbital blood collection were analyzed for ALT, AST, CRE, LDH, CK-MB, and urea levels. Tumor volume was calculated using the formula: Tumor volume (mm^3^) = A × B^2^ × 0.5, where A represents the longest diameter and B the shortest diameter. The tumor inhibition rate was determined based on the following equation:

$$\text{Tumor}\;\text{inhibition}\;\text{ratio}\;\left(\%\right)=\frac{W_{N}-W_{s}}{w_{N}}\times 100\%$$

where W_N_ and W_s_ represent the mean tumor weights in the control (normal saline) and treatment groups, respectively.

### 5.26 Immunohistochemistry

Tumor xenograft tissues were fixed in 4% paraformaldehyde, embedded in paraffin, and sectioned onto positively charged slides. Following deparaffinization and antigen retrieval, the sections were incubated overnight at 4 °C with primary antibodies. Subsequently, the sections were incubated with corresponding secondary antibodies and developed using 3,3′-diaminobenzidine (DAB) chromogen according to standard protocols. All images were acquired using a DM2500 bright-field microscope (Leica Microsystems, Wetzlar, Germany).

### 5.27 Statistical analysis

All statistical analyses were performed using GraphPad Prism 8.0 (GraphPad Software, Inc., La Jolla, CA, USA). Data are presented as mean ± standard deviation (SD). Comparisons among multiple groups were performed using one-way analysis of variance (ANOVA). Statistical significance was defined as *P < 0.05, **P < 0.01, ***P < 0.001, and NS indicates not significant.

## Ethics approval and consent to participate

All *in vivo* experiments and animal procedures were conducted in accordance with the animal protection guidelines of Sichuan University (SYXK(Sichuan)2019-0005) and the approved guidelines of the Institutional Animal Care and Use Committee of Sichuan University.

## Consent for publication

All authors give consent for the publication of the manuscript in *Materials Today Bio*.

## Availability of data and materials

Upon reasonable request, the corresponding author will provide the data that backs up the study's conclusions.

## Funding

This work was supported by the National Key Research and Development Project (No. 2023YFC3402100), the 10.13039/501100001809National Natural Science Foundation of China (NSFC: 823B2081, 82130082, 82303838), the 10.13039/501100004829Sichuan Provincial Department of Science and Technology (2025ZNSFSC0001), the Postdoctoral Fellowship Program, the 10.13039/501100002858China Postdoctoral Science Foundation (Grant No. BX20250245), the Sichuan Science and Technology Program (2025ZNSFSC1888), the Open Research Fund Program of the Key Laboratory of Xinjiang Endemic and Ethnic Disease (KF202403) and the Science and Technology Development Program of Jilin Province (20260203011SF).

## CRediT authorship contribution statement

**Shanshan Liu:** Data curation, Formal analysis, Methodology, Software, Writing – original draft. **Yichun Huang:** Data curation, Formal analysis, Methodology, Software, Writing – original draft. **Fanchen Yan:** Data curation, Formal analysis, Methodology, Software, Writing – original draft. **Hailong Tian:** Writing – review & editing. **Bowen Li:** Formal analysis. **Yaying Zhang:** Software. **Huili Zhu:** Investigation, Validation, Writing – review & editing. **Weihua Tong:** Supervision, Validation, Writing – review & editing. **Canhua Huang:** Conceptualization, Funding acquisition, Project administration, Resources, Supervision, Writing – review & editing.

## Declaration of competing interest

The authors declare that they have no known competing financial interests or personal relationships that could have appeared to influence the work reported in this paper.

## Data Availability

Data will be made available on request.
