# Supplementary material for: Cuproptosis-mediated stemness inhibition by a self-assembled herbal nanoplatform overcomes chemoresistance in ovarian cancer
Source: Mater Today Bio. 2026 Jul 9;39:103440. doi: 10.1016/j.mtbio.2026.103440 (PMC13380750; doi:10.1016/j.mtbio.2026.103440)
Supplement: Multimedia component 1 [file mmc1.docx]

**Supporting Information**

**Cuproptosis-Mediated Stemness Inhibition by a Self-Assembled Herbal Nanoplatform Overcomes Chemoresistance in Ovarian Cancer**

Shanshan Liu^a,1^, Yichun Huang^b,1^, Fanchen Yan^c,1^Hailong Tian^d^, Bowen Li^d^, Yaying Zhang^d^, Huili Zhu^e,*^, Weihua Tong^f,*^, Canhua Huang^a,d,g,**^

*^a^School of Health Preservation and Rehabilitation, Chengdu University of Traditional Chinese Medicine, Chengdu, 611137, China.*

*^b^Department of Surgical Oncology, The First Affiliated Hospital of Kunming Medical University, Kunming, 650032, China*

*^c^School of Basic Medical Sciences, Chengdu University of Traditional Chinese Medicine, Chengdu, 611137, China*

*^d^Department of Biotherapy, Oxidative Stress Research Center, Cancer Center and State Key Laboratory of Biotherapy, West China Hospital, Sichuan University, Chengdu, Sichuan, 610041, China.*

*^e^Department of Reproductive Medicine, Key Laboratory of Birth Defects and Related Diseases of Women and Children of Ministry of Education, West China Second University Hospital of Sichuan University, Chengdu, Sichuan, 610041, China.*

*^f^Obstetrics and Gynecology Center, The First Hospital of Jilin University, Changchun, Jilin, 130012, China.*

*^g^Frontiers Medical Center, Tianfu Jincheng Laboratory, Chengdu, 610041, P.R. China.*

^1^*Authors contributed equally.*

^*^Corresponding authors

E-mail: hlzhu78@139.com; tongwh@jlu.edu.cn; hcanhua68@163.com;

**Table Legend**

**Table S1**. Primary antibodies in this study.

| **Antibody name** | **Company and Cat. No.** |
| --- | --- |
| Hsp70 | Abcam, ab181606 |
| β-Actin | ABclonal, AC026 |
| c-Myc | ABclonal, A19032 |
| Nanog | ABclonal, A22625 |
| Oct4 | Abcam, ab19857 |
| Lipoic acid | Abcam, ab58724 |
| KLF4 | Abways, CY6648 |
| DLAT | Cell Signaling Technology, # 12362S |
| Ki67 | Cell Signaling Technology, #9449 |
| CD133 | Proteintech, 18470-I-AP |
| FDX1 | Proteintech, 12592-1-AP |
| LIAS | Proteintech, 67298-1 |
| SOX2 | ZEN BIO, 200146 |

**Table S2**. Assay Kits in this study.

| **Assay Kit** | **Company and Cat. No.** |
| --- | --- |
| Reactive Oxygen Species Assay Kit | Beyotime, S0033M |
| LDH Cytotoxicity Assay Kit | Beyotime, C0017 |
| ATP Assay Kit | Beyotime, S0026 |
| Annexin V-FITC Apoptosis Detection Kit | Livning, 556547 |
| Mitochondrial membrane potential assay kit with JC-1 | Beyotime, C2006 |
| Cell-Light^TM^ EdU Apollo In Vitro Kit | RIBOBIO, C10310-1 |
| Cell Counting Kit-8 | Beyotime, C0039 |
| GSH and GSSG Assay Kit | Beyotime, S0053 |
| Glutathione Reductase Assay Kit with DTNB | Beyotime, S0055 |
| Copper (Cu^2+^) Colorimetric Assay Kit | Elabscience, E-BC-K300-M |

**Table S3**. Chemical reagents in this study.

| **Chemical reagents** | **Company and Cat. No.** |
| --- | --- |
| Shikonin | Meilunbio Co., Ltd. MB7082 |
| Cu(NO3)_2_3H_2_O | Aladdin Reagent Co., Ltd. 10031-43-3 |
| Doxorubicin hydrochloride | Aladdin Reagent Co., Ltd. 25316-40-9 |
| Bathocuproine | Aladdin Reagent Co., Ltd. B110336 |
| Rotenone | Aladdin Reagent Co., Ltd. R426175 |
| Elesclomol | MedChemExpress, Co., Ltd. 488832-69-5 |
| Sodium hydroxide (NaOH) | Aladdin Reagent Co., Ltd. S111518 |
| Hyaluronic acid (HA) | Meilunbio Co., Ltd. MB12810 |
| Dimethyl sulfoxide (DMSO) | Aladdin Reagent Co., Ltd. D755829 |


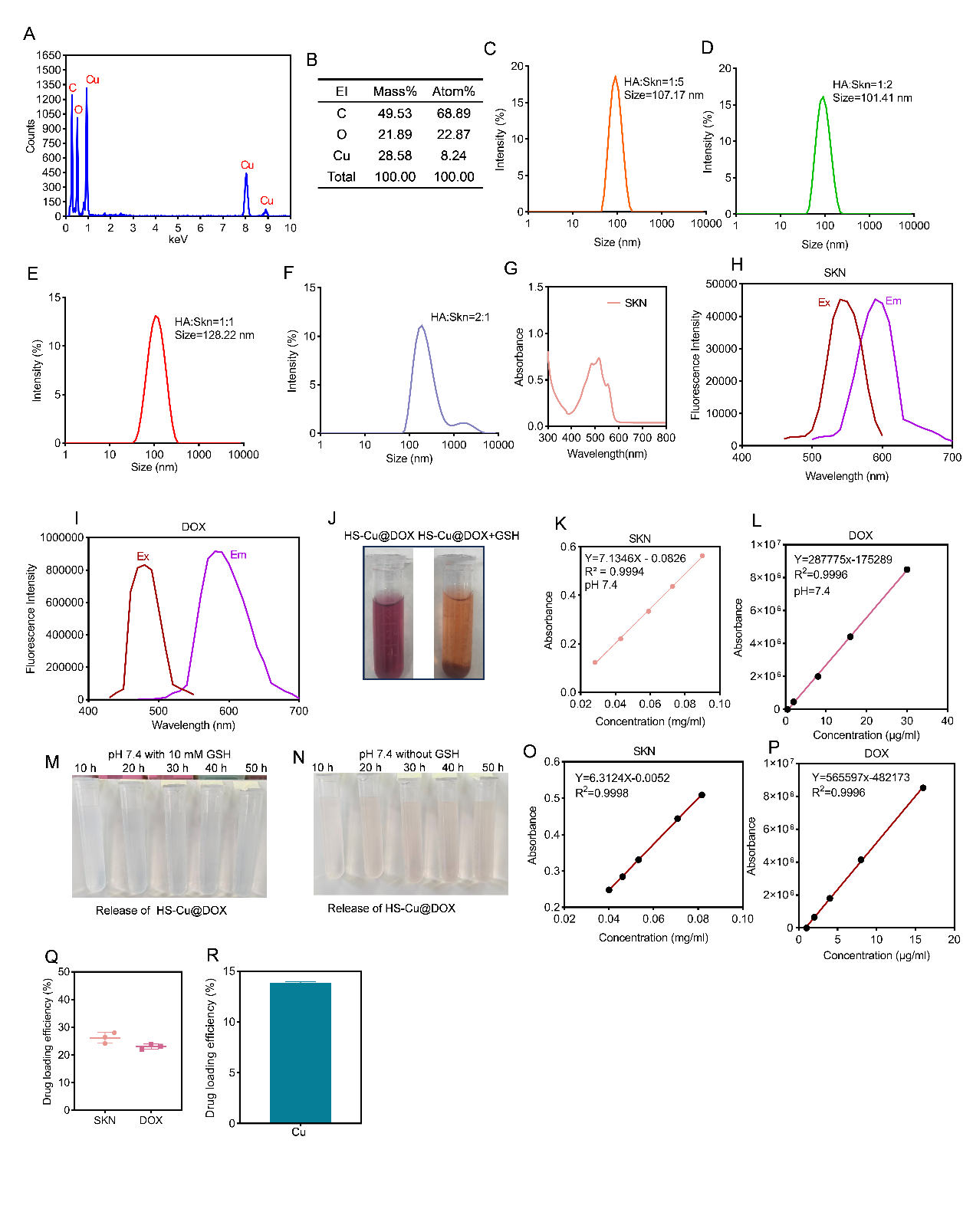


**Figure S1.** Preparation and characterization of HS-Cu@DOX. A) EDS spectra of S-Cu. B) Elemental analysis of S-Cu. C-F) Size distribution of HS-Cu at different mass ratios of HA and SKN. G) UV−vis spectra of SKN in ethanol. H-I) Fluorescence characterization of (H) SKN and DOX (I). J) The picture of HS-Cu@DOX after GSH treatment. K, L) Standard curves of SKN and DOX at pH values of 7.4. M) Representative photographs of HS-Cu@DOX nanoparticle dispersions collected at different time points during incubation in PBS (pH 7.4) containing 10 mM GSH. N) Representative photographs of HS-Cu@DOX nanoparticle dispersions collected at different time points during incubation in PBS (pH 7.4) without GSH. O) Standard curve of SKN. P) Standard curve of DOX was established by HPLC.Q) The SKN and DOX loading efficiency of HS-Cu@DOX (n = 3). R) Cu²⁺ loading efficiency of HS-Cu@DOX determined by ICP-OES (n = 3).


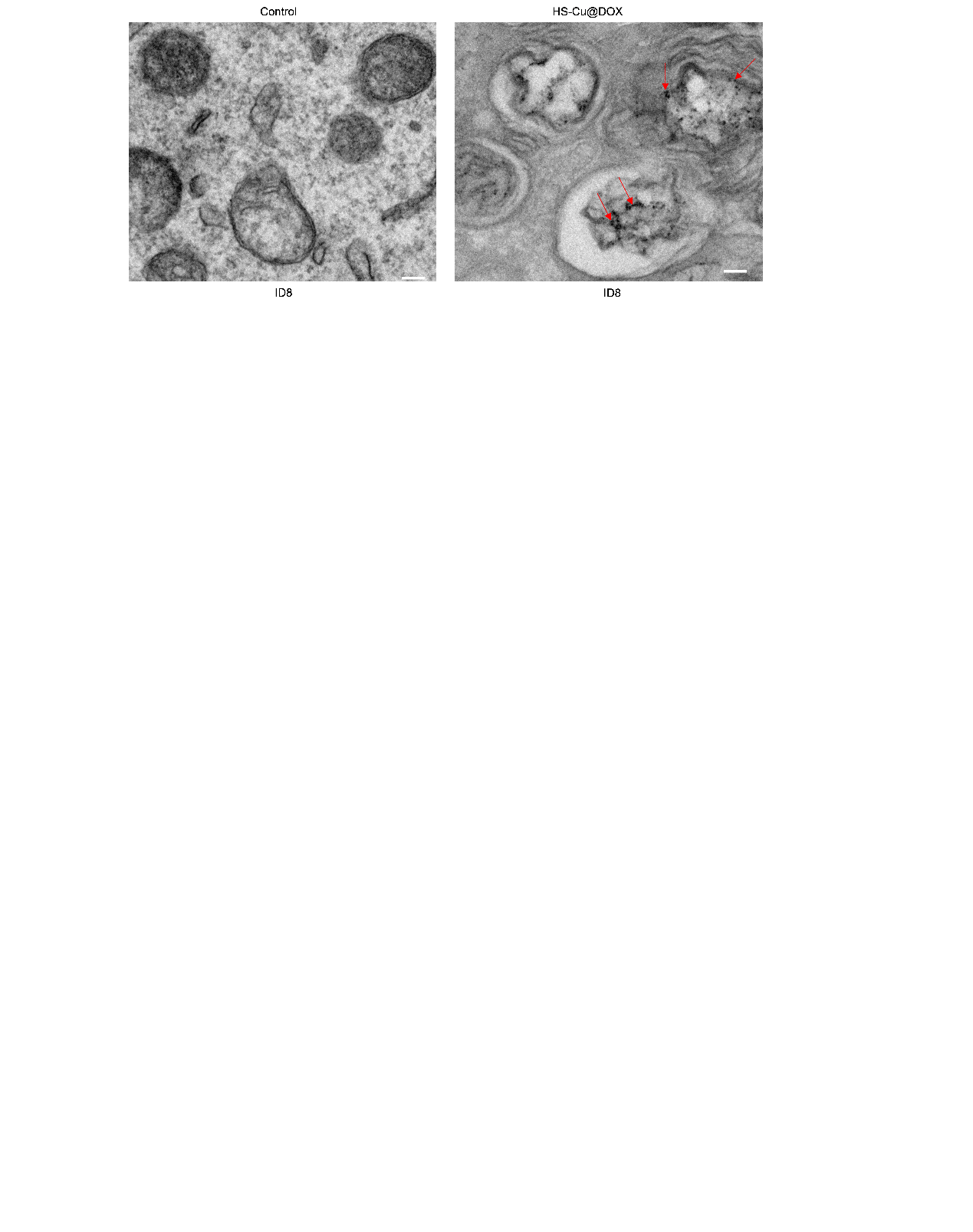


Figure S2. Transmission electron microscopy (TEM) images of ID8 cells under different conditions. Scale bar: 1 μm.


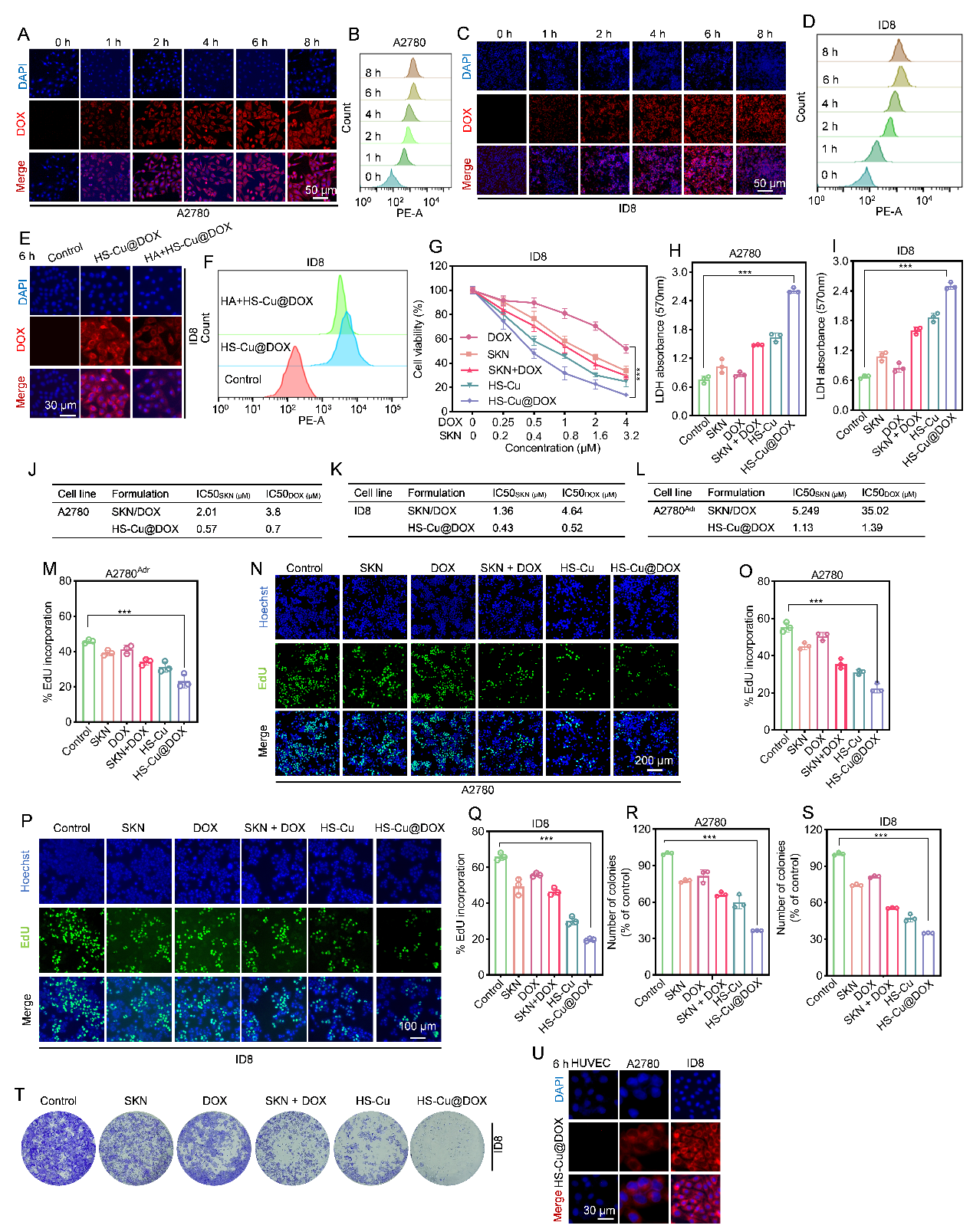


**Figure S3.** The cellular internalization and cytotoxic effects of HS-Cu@DOX *in vitro*. A-D) HS-Cu@DOX uptake in A2780 and ID8 cells evaluated by (A, C) fluorescence microscopy and (B, D) flow cytometry (n=3). Scale bar: 50 µm. E, F) Cellular uptake of Control, HS-Cu@DOX and HA + HS-Cu@DOX in ID8 cells after 6 h incubation, as examined by fluorescence microscopy and flow cytometry. Cells were pretreated with HA for 30 min. Scale bar: 30 µm. G) Cell viability of ID8 cells following treatment with Control, DOX, SKN, DOX+SKN, HS-Cu and HS-Cu@DOX (n = 3). H, I) LDH assay of A2780 and ID8 cells co-cultured with Control, DOX, SKN, DOX+SKN, HS-Cu and HS-Cu@DOX. J-L) IC50 of SKN and DOX in different groups of A2780 (J), A2780^Adr^ (K), and ID8 (L) cells. M) Quantitative analysis of A2780^Adr^ cells cocultured with Control, DOX, SKN, DOX+SKN, HS-Cu and HS-Cu@DOX in the EdU assay. N-Q) Fluorescent microscope images and quantitative analysis of A2780 and ID8 cells cocultured with Control, DOX, SKN, DOX+SKN, HS-Cu and HS-Cu@DOX in the EdU assay. Scale bar: 200 µm. R) the statistical analysis for colony formation of A2780 cells following treatment with DOX, SKN, DOX+SKN, HS-Cu and HS-Cu@DOX. (n = 3) S, T) The statistical analysis and images for colony formation of ID8 cells following treatment with DOX, SKN, DOX+SKN, HS-Cu and HS-Cu@DOX. U) Cellular uptake of HS-Cu@DOX in HUVEC, A2780, and ID8 cell lines. Scale bar: 30 µm. (****P* < 0.001, one-way ANOVA).


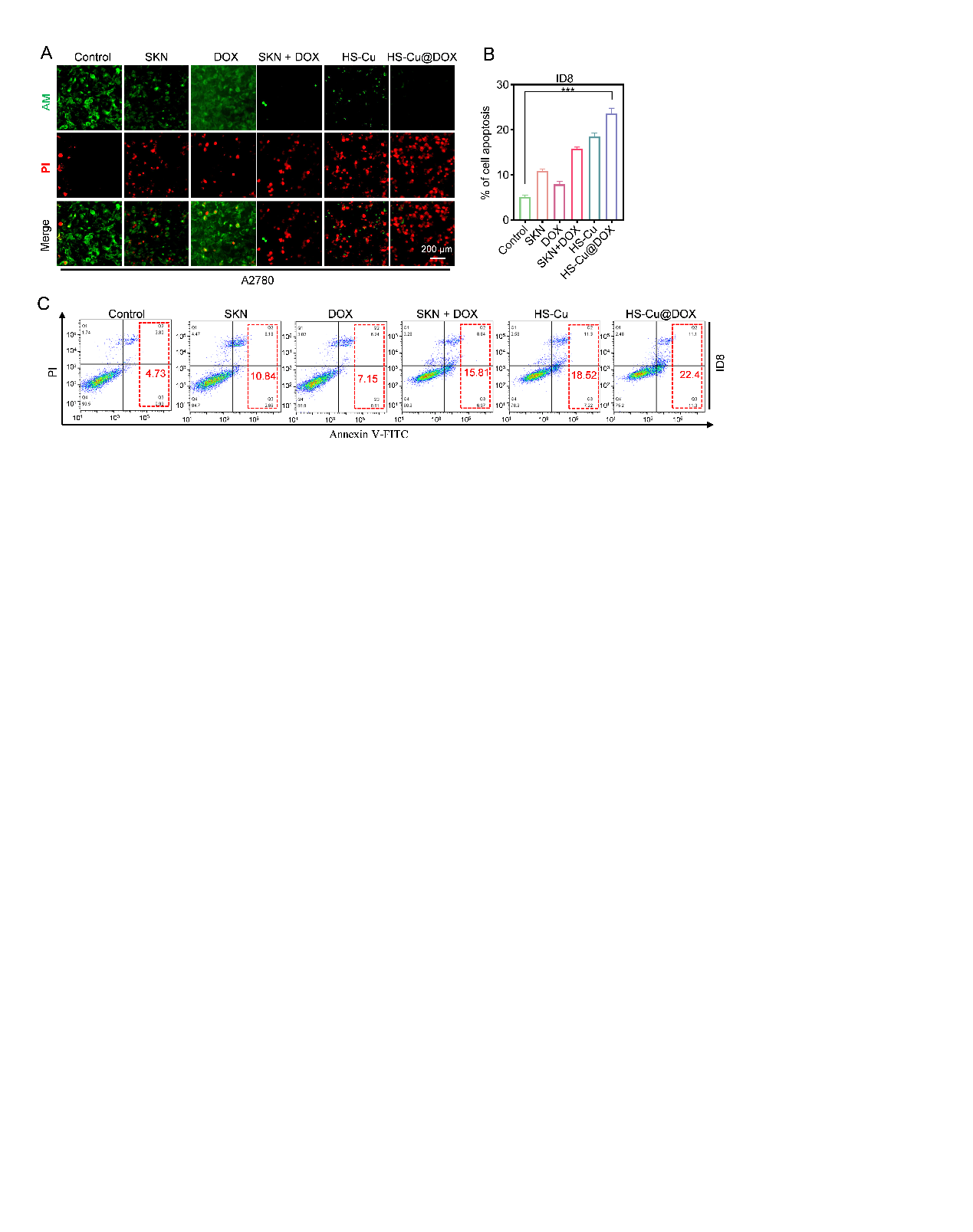


**Figure S4.** The cellular internalization and cytotoxic effects of HS-Cu@DOX *in vitro*. A) Fluorescence imaging of AM/PI staining assay in A2780 cells with different treatments; scale bar:200μm. B, C) Flow cytometric analysis and quantification of apoptosis in ID8 cells after various treatments using Annexin V-FITC/PI staining. (****P* < 0.001, one-way ANOVA).


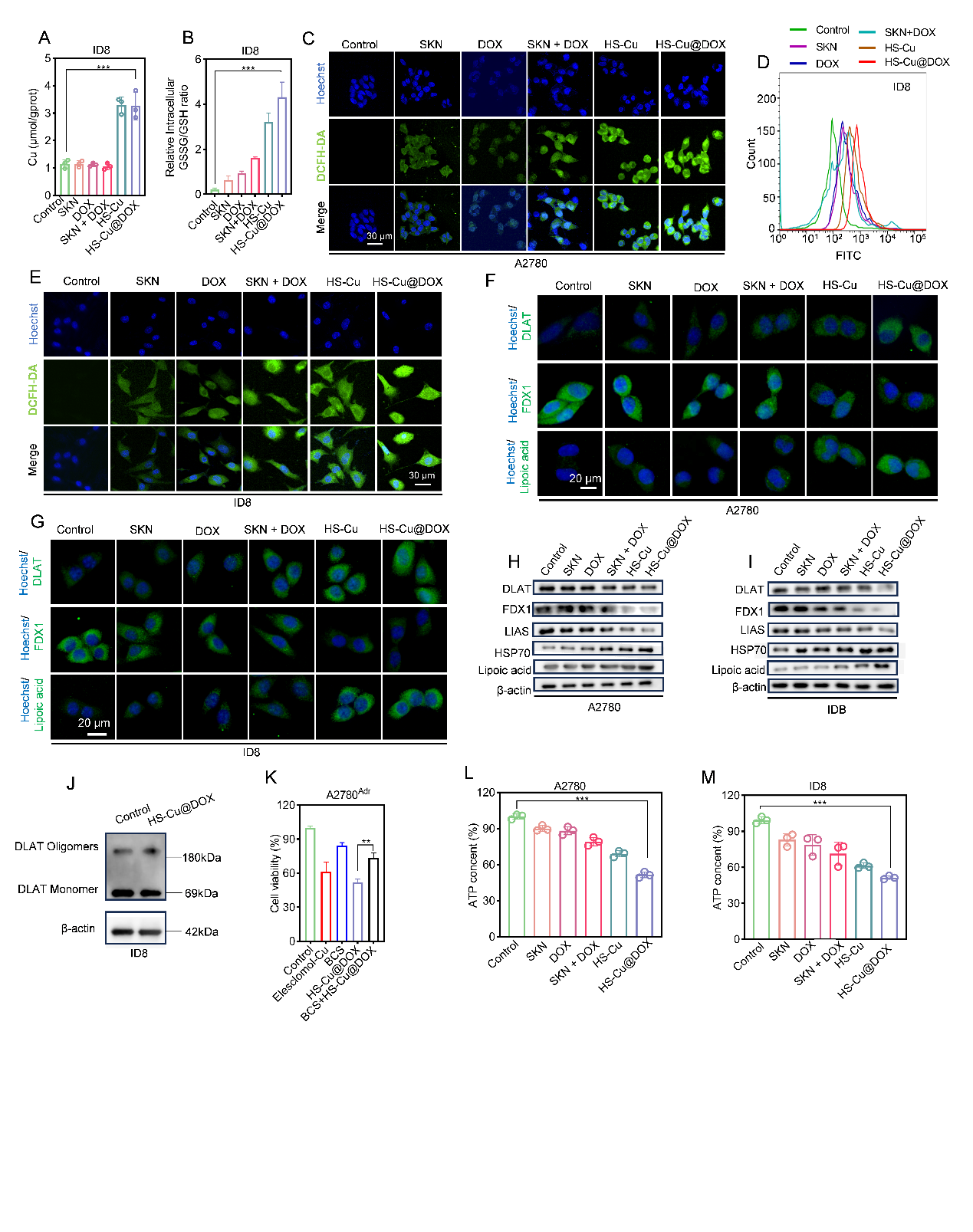


**Figure S5.** HS-Cu@DOX induced cuproptosis through mitochondrial copper overload and metabolic collapse. A) Intracellular copper levels in ID8 cells after various treatments (n = 3, ****P* < 0.001, one-way ANOVA). B) The ratio of GSSG/GSH in ID8 cells was measured following different treatments (n = 3, ****P* < 0.001, one-way ANOVA). C) The fluorescence images of ROS generation in A2780 cells with various treatments. (Scale bar:30 µm). D-E) The flow cytometry analysis and fluorescence images of ROS generation in ID8 cells with various treatments. (Scale bar:30 µm). F-G) Fluorescence images of DLAT, FDX1 and Lipoic acid of A2780 (F) and ID8 (G) cells with the indicated treatments. (Green channel, DLAT, FDX1 and Lipoic acid; blue channel, DAPI; scale bar: 30 µm). H-I) Immunoblotting analysis of DLAT, FDX1, LIAS, HSP70, and lipoic acid in A2780 and ID8 cells treated with different reagents. J) Western blot analysis of cuproptosis-associated DLAT monomers and oligomers in ID8 ovarian cancer cells. K) Cell viability was detected in A2780^Adr^ cells with elesclomol (copper inducer) and BCS (copper chelator) to verify HS-Cu@DOX-triggered cuproptosis. L, M) Intracellular ATP secretion levels of A2780 and ID8 cells under various conditions. (n=3)


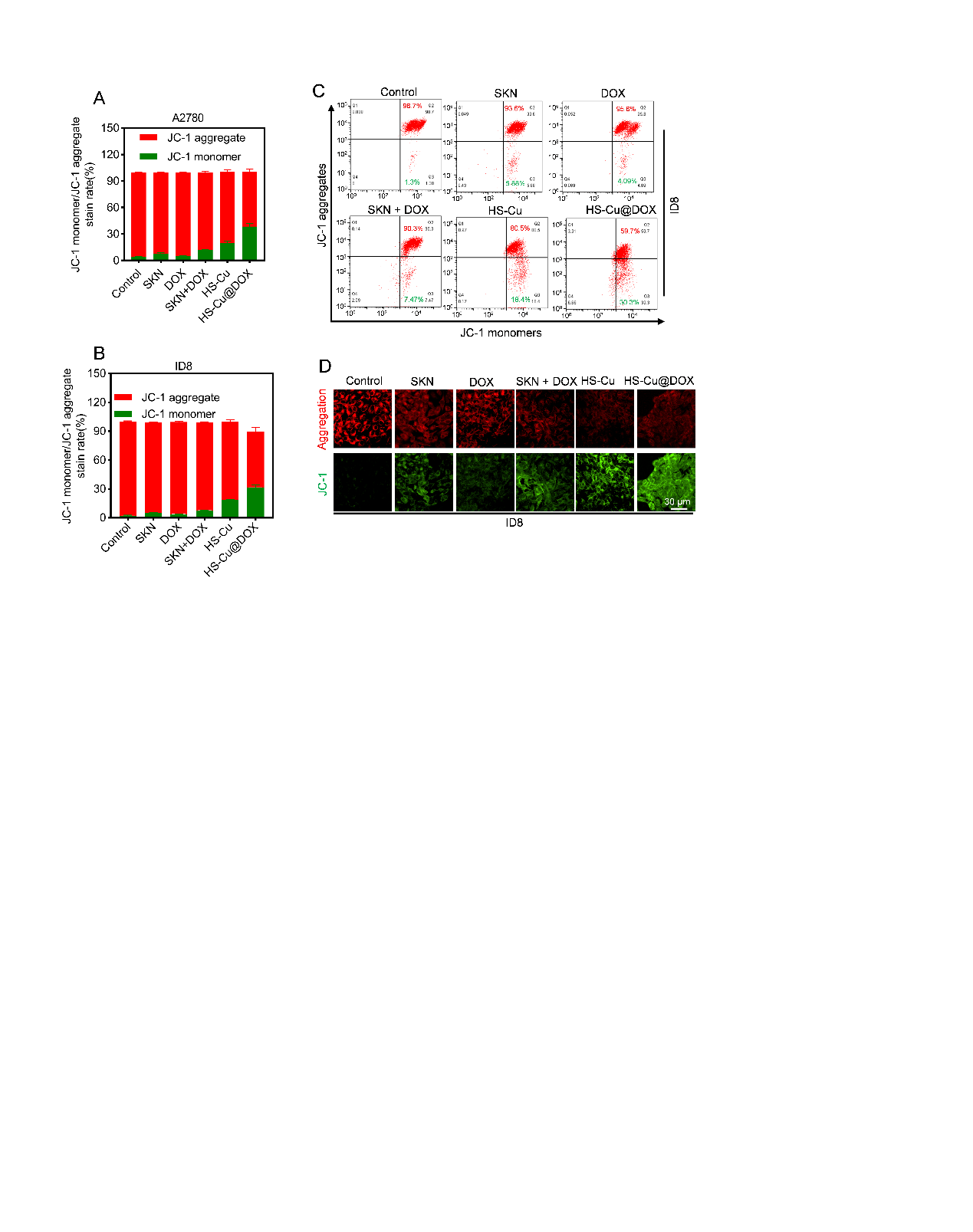


**Figure S6.** HS-Cu@DOX induced cuproptosis through mitochondrial copper overload and metabolic collapse. C) JC-1 flow cytometry of ID8 cells. A, B) Quantitative analysis of ΔΨm in A2780 and ID8 cells. D) The JC-1 fluorescence graph in ID8 cells treated with various treatments. (n=3; ****P* < 0.001; one-way ANOVA).


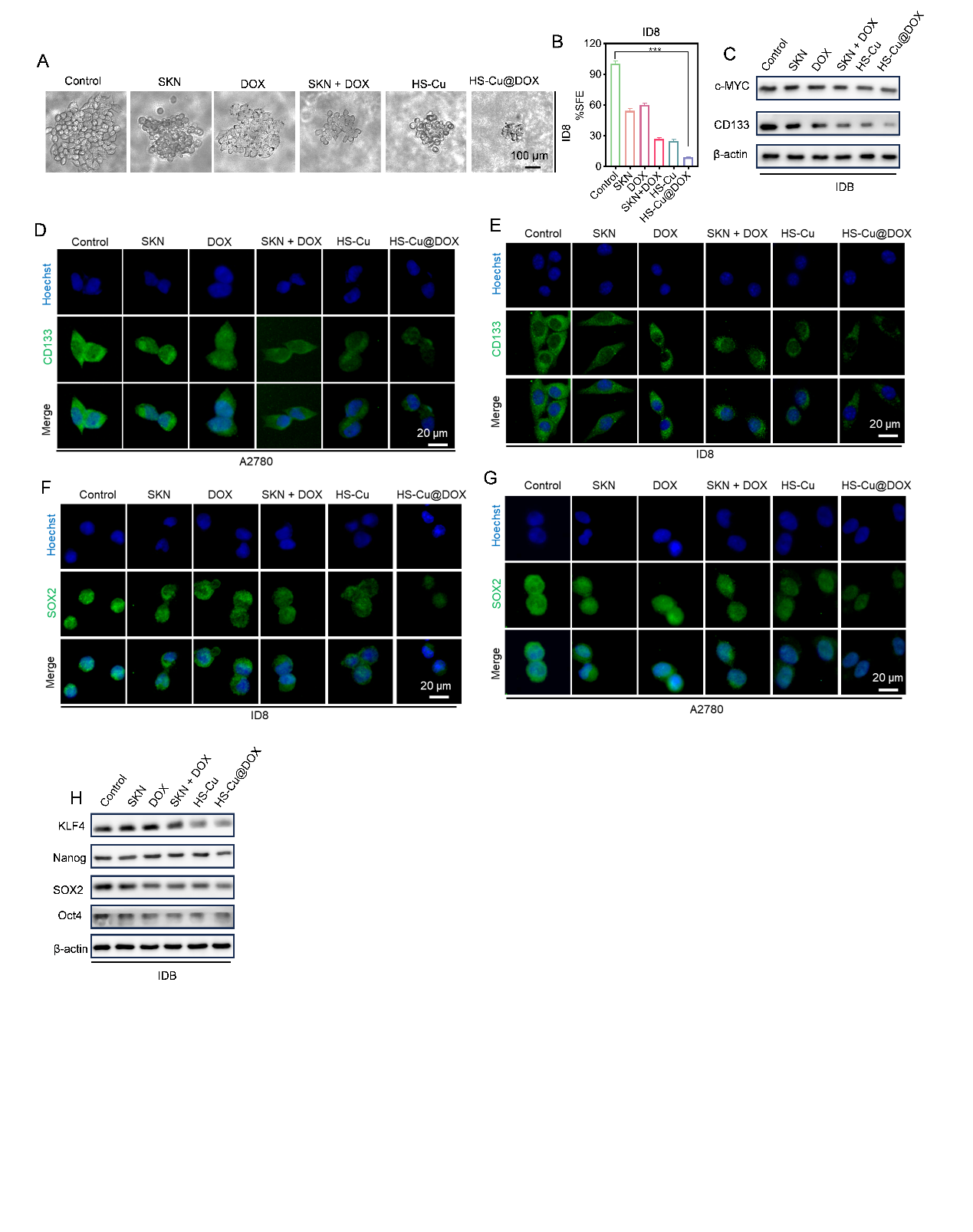


**Figure S7.** HS-Cu@DOX Overcomes Chemoresistance by Suppressing Cancer Stemness through Cuproptosis-Dependent Mechanisms. A-B) Images and statistical analysis of SFE with SKN, DOX, SKN+DOX, HS-Cu, and HS-Cu@DOX. Scale bar: 50 μm. (n=3; ****P* < 0.001; one-way ANOVA) C) Representative western blotting of CD133 and c-Myc proteins in ID8 cells. D-E) Immunofluorescence analysis of CD133 protein in A2780 and IDB cells treated with SKN, DOX, SKN+DOX, HS-Cu, and HS-Cu@DOX. Scale bar = 20 μm. F-G) Immunofluorescence analysis of SOX2 protein in ID8 and A2780 cells treated with SKN, DOX, SKN+DOX, HS-Cu, and HS-Cu@DOX. Scale bar = 20 μm. H) Expression of KLF4, Nanog, SOX2, OCT4, proteins in ID8 cells using western blotting assay.


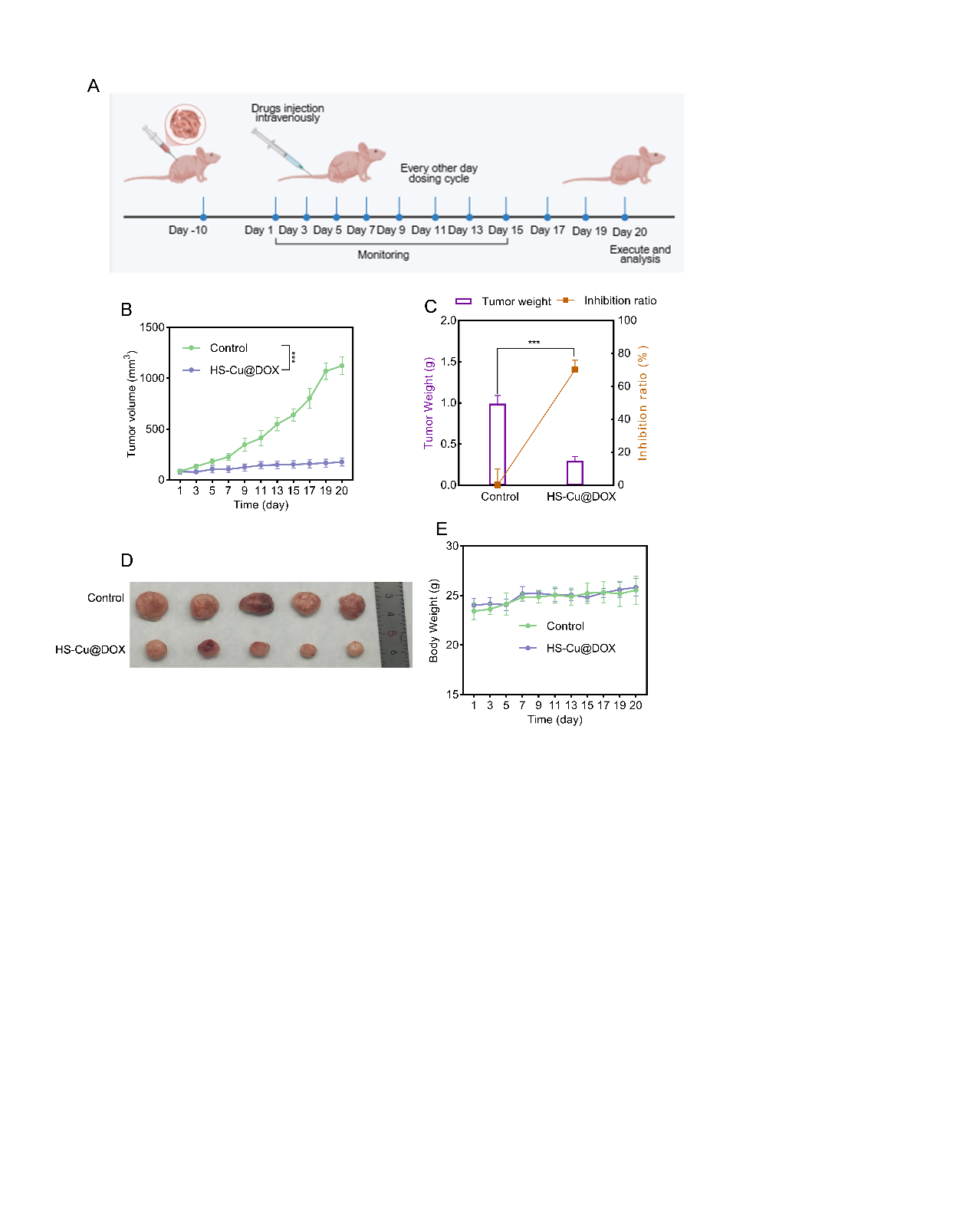


Figure S8. A) Schematic of the A2780 tumor therapy regimen in BALB/c nude mice. Created with BioRender.com. B) Tumor volume by group at designated time points (n = 5; **P < 0.01; one-way ANOVA). C) Tumor weight and inhibition rate by group (n = 5; ***P < 0.001; one-way ANOVA). D) Images depicting individual tumors (n = 5). E) Body weight of mice in different groups (n = 5).
